# Food matters: Dietary shifts increase the feasibility of 1.5°C pathways in line with the Paris Agreement

**DOI:** 10.1126/sciadv.adj3832

**Published:** 2024-03-27

**Authors:** Florian Humpenöder, Alexander Popp, Leon Merfort, Gunnar Luderer, Isabelle Weindl, Benjamin Leon Bodirsky, Miodrag Stevanović, David Klein, Renato Rodrigues, Nico Bauer, Jan Philipp Dietrich, Hermann Lotze-Campen, Johan Rockström

**Affiliations:** ^1^ Potsdam Institute for Climate Impact Research (PIK), Member of the Leibniz Association, Potsdam, Germany.; ^2^ Faculty of Organic Agricultural Sciences, University of Kassel, Witzenhausen, Germany.; ^3^ Global Energy Systems Analysis, Technische Universität Berlin, Berlin, Germany.; ^4^ Humboldt University of Berlin, Berlin, Germany.; ^5^ Institute of Environmental Science and Geography, University of Potsdam, Potsdam, Germany.

## Abstract

A transition to healthy diets such as the EAT-Lancet Planetary Health Diet could considerably reduce greenhouse gas (GHG) emissions. However, the specific contributions of dietary shifts for the feasibility of 1.5°C pathways remain unclear. Here, we use the open-source integrated assessment modeling (IAM) framework REMIND-MAgPIE to compare 1.5°C pathways with and without dietary shifts. We find that a flexitarian diet increases the feasibility of the Paris Agreement climate goals in different ways: The reduction of GHG emissions related to dietary shifts, especially methane from ruminant enteric fermentation, increases the 1.5°C compatible carbon budget. Therefore, dietary shifts allow to achieve the same climate outcome with less carbon dioxide removal (CDR) and less stringent CO_2_ emission reductions in the energy system, which reduces pressure on GHG prices, energy prices, and food expenditures.

## Teaser

A global transition to healthy diets cuts 1.5°C compatible GHG prices in 2050 by 43% compared to continued dietary trends.

## INTRODUCTION

Food system transformation toward sustainable healthy diets is the most effective demand-side option to mitigate climate change, according to the Sixth Assessment Report (AR6) of the United Nation’s Intergovernmental Panel on Climate Change (IPCC) (*1*). The Planetary Health Diet, as proposed by the EAT-Lancet Commission on healthy diets from sustainable food systems, entails a marked reduction of livestock products such as meat and milk, primarily in middle- and high-income regions, in favor of vegetables, fruits, nuts, and legumes (*2*). The indicative global mitigation potential of sustainable healthy diets could reach 7 GtCO_2_-eq year^−1^ by 2050 (corresponding to about one-third of total food system emissions) if land-use change and re-/afforestation of freed up land are considered in addition to lower methane (CH_4_) and nitrous oxide (N_2_O) emissions from agriculture (*1*). Moreover, a shift to healthy diets could considerably lower the impacts of food production on other sustainability dimensions such as water, nitrogen, and biodiversity (*3*–*6*), which in turn could reduce the economic costs related to human health and ecosystem degradation (*7*).

The mitigation potential of dietary shifts within the land system has been analyzed in numerous studies (*8*–*13*). However, the dynamic interplay of dietary shifts in the land system with the decarbonisation of the energy system in a Paris Agreement 1.5°C transformation pathway has been rarely analyzed (*3*, *14*, *15*). In these studies, dietary shifts have been combined with a larger portfolio of mitigation options (e.g., modal shift, electrification of transport, or technical on-farm measures) or optimistic assumptions regarding future population growth and economic development [based on the sustainability focused Shared Socio-economic Pathway 1 (SSP1)]. Therefore, the existing literature does not allow to single out the contribution of dietary shifts alone for the feasibility of 1.5°C in terms of remaining carbon budget; required carbon dioxide removal (CDR); speed of energy system decarbonisation; and implications for carbon prices, energy prices, and food expenditures. Considering the prominent role of dietary shifts in the scientific literature and public discourse (*10*, *16*–*18*), we specifically aim to investigate in this study the contribution of dietary shifts toward the feasibility of 1.5°C transformation pathways.

### Study setup

We use the open-source integrated assessment modeling (IAM) framework REMIND-MAgPIE (REMIND 3.2.0 and MAgPIE 4.6.7; see figs. S1 to S4 for an overview) for contrasting three transformation pathways, all based on SSP2 middle-of-the-road assumptions for population, income, and other key drivers for land and energy systems (Table 1 and Materials and Methods). SSP2 is characterized by the continuation of current trends for population and income into the future (see figs. S8 and S9 for comparison with other SSPs). Global population peaks at 9.75 billion people in 2070, largely because of slow demographic transition in low-income countries (fig. S5A). At the same time, income increases in all countries but inequality gaps between countries remain largely unresolved (fig. S5B).

**Table 1.** Key scenario assumptions in REMIND-MAgPIE. The three pathways SSP2-NDC, SSP2-1.5°C, and SSP2-1.5°C-DietShift follow SSP2 parametrizations, which reflect a continuation of current socioeconomic trends into the future. Population and income are important model drivers for energy and food demands. SSP2 population and income trajectories reflect middle-of-the-road assumptions compared to more optimistic (SSP1 sustainability) and more pessimistic (SSP3 regional rivalry) trajectories (see figs. S8 and S9). The qualitative descriptions for population and income are based on the numbers shown in fig. S5. The qualitative descriptions for per-capita calorie intake and dietary composition are based on the numbers shown in fig. S6. NDCs are national climate action plans under the Paris Agreement. The peak carbon budget limits the remaining global cumulative CO_2_ emissions across the energy and land system until net-zero annual CO_2_ emissions must be reached.

	SSP2-NDC	SSP2-1.5°C	SSP2-1.5°C-DietShift
Population	Global population peaks at 9.75 billion people in 2070, largely driven by population growth in low-income countries		
Income	Income increases in all countries but inequalities between countries remain largely unresolved		
Per-capita calorie intake	Too high in high- and middle-income countries between 2020 and 2050, strong increase in low-income countries from insufficient levels in 2020 to unbalanced levels in 2050		Transition to balanced intake levels corresponding to a healthy BMI by 2050 in all countries
Dietary composition	Livestock share in high-and middle-income countries remains at high levels, strong increase of livestock share in low-income countries by 2050		Transition to EAT-Lancet Planetary Health Diet (flexitarian diet) by 2050 in all countries
Food waste	No dedicated measures for food waste reduction. Food waste scales proportional to food intake for each time step.		
Climate policy in energy system	Carbon price and modern bioenergy demand are based on NDCs	Consistent GHG emission prices across sectors and GHGs (in units of USD2020 per metric ton CO_2_-eq) and levels of modern bioenergy across land and energy systems are endogenously derived in an iterative approach by imposing the respective peak carbon budget. Resulting GHG prices are applied on all energy system emissions and from 2035 onward also on all GHG emissions in the land system. In addition, land- and energy-sector–based NDCs are considered.	
Climate policy in land system	Land-based NDCs for reduced deforestation and reforestation		
Peak carbon budget for land and energy system	–	500 Gt CO_2_ from 2020 onward	625 Gt CO_2_ from 2020 onward
Climate impacts on crop yields, water and carbon	RCP 4.5 4.5 W/m^2^ in 2100	Representative Concentration Pathway (RCP) 1.9 stabilizes radiative forcing at 1.9 W/m^2^ in the year 2100	

The first pathway, SSP2-NDC, includes so-called nationally determined contributions (NDCs) as climate policies with greenhouse gas (GHG) emission reduction targets across different sectors of the economy. NDCs are national climate action plans under the Paris Agreement. All 195 countries that signed the Paris Agreement were required to submit an initial NDC, which should be updated every 5 years, depending on the ambition and implementation gap (ratcheting-up mechanism). In addition, countries are invited to submit their long-term low-emission development strategies. In this study, we consider NDCs for 2030 including extrapolation until 2100 but no long-term targets. Previous studies have shown that NDCs for 2030 alone are insufficient to meet the Paris Agreement goal of limiting global warming to 1.5°C (*19*).

The second pathway, SSP2-1.5°C, includes 1.5°C compatible climate policies in the energy and land system, in addition to NDCs. GHG prices and deployment of modern bioenergy in line with the Paris Agreement 1.5°C goal are endogenously derived by an iterative soft coupling of the energy-economy model REMIND and the land-use model MAgPIE (see Materials and Methods for details). A so-called peak carbon budget of 500 GtCO_2_ from 2020 onward limits the remaining global cumulative CO_2_ emissions across the energy and land system until net-zero annual CO_2_ emissions must be reached. The taxing of GHG emissions in the energy and land system, hereafter referred to as GHG emission pricing, is the main policy instrument to meet this target.

The third pathway, SSP2-1.5°C-DietShift, has the same setup as SSP2-1.5°C but includes dietary shifts toward the EAT-Lancet Planetary Health Diet by 2050 in all world regions. The EAT-Lancet Planetary Health Diet is a flexitarian diet predominantly featuring a wide variety of plant-based foods, limited consumption of animal-derived foods, a preference for unsaturated fats over saturated ones, and restricted intake of highly processed foods and added sugars. In addition, per-capita calorie intake converges to levels in line with a healthy body mass index (BMI) by 2050 in all world regions. To single out the effect of these dietary shifts, no dedicated measures for food waste reduction are included in this study. The reduction of GHG emissions related to dietary shifts, especially short-lived methane from ruminant enteric fermentation, would result in a lower peak temperature increase than in SSP2-1.5°C. For comparability in terms of climate outcome, the SSP2-1.5°C-DietShift pathway is designed to match the peak temperature increase of the SSP2-1.5°C pathway via an increased carbon budget of 625 Gt CO_2_ from 2020 onward.

## RESULTS

### Food demand trajectory

**Fig. 1.**
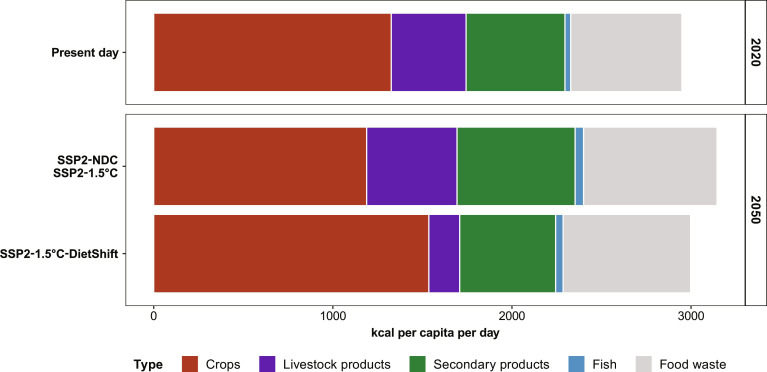
Overview of scenario assumptions for dietary composition, per-capita food intake and food waste. Data are shown at global level for the years 2020 and 2050. In SSP2-NDC and SSP2-1.5°C, dietary composition follows SSP2 assumptions and there is no target for per-capita intake levels. In SSP2-1.5°C-DietShift, dietary composition converges to the EAT-Lancet Planetary Health Diet (flexitarian diet) in all world regions by 2050. At the same time, per-capita calorie intake converges to levels in line with a healthy BMI in all world regions by 2050. Crops, livestock products, secondary products, and fish reflect per-capita calorie intake levels. Food waste is the aggregate of waste from all food intake categories. Thus, the sum of intake and food waste levels equals per-capita food supply. See figs. S6 and S12 for regional figures.

Population and income follow SSP2 trajectories in all three pathways (figs. S5, A and B; S8; and S9). Per-capita calorie intake increases in SSP2-NDC and SSP2-1.5°C in low-, middle-, and high-income regions throughout the 21st century based on SSP2 assumptions (fig. S5C). In SSP2-1.5°C-DietShift, per-capita calorie intake declines in high- and middle-income regions throughout the 21st century but increases in low-income regions toward a balanced intake level for a healthy BMI by 2050, also considering the demographic structure of each country (fig. S5C; see fig. S10 for regional figures also accounting for food waste). Food waste is very similar in all three pathways (Fig. 1 and fig. S11) because no dedicated measures for food waste reduction are included in this study, which specifically focuses on dietary shifts. EAT-Lancet recommendations for the composition of a flexitarian diet entail a marked reduction of livestock products in favor of plant-based products, especially in high- and middle-income regions (fig. S6; see table S1 for regional definitions and fig. S12). On global average, per-capita intake of livestock products declines from 418 to 172 kcal per cap per day between 2020 and 2050. At the same time, intake of crops increases from 1326 to 1536 kcal per cap per day by 2050. Combined with population, the resulting total global demand for crops and livestock products (food, feed, and other purposes) throughout the 21st century in SSP2-1.5°C-DietShift is almost constant over time, in contrast to an increasing trend in SSP2-NDC and SSP2-1.5°C (>50% higher in 2100; figs. S5D and S13)*.* The prevalence of underweight slightly declines in SSP2-NDC and SSP2-1.5°C. Calorie intake in line with a healthy BMI in the SSP2-1.5°C-DietShift pathway reduces prevalence of underweight to zero by 2050 (fig. S5E). A healthy caloric intake also eliminates obesity by 2050 in SSP2-1.5°C-DietShift, while obesity strongly increases under SSP2 assumptions (fig. S5F).

### Dietary shifts increase the feasibility of 1.5°C pathways

**Fig. 2.**
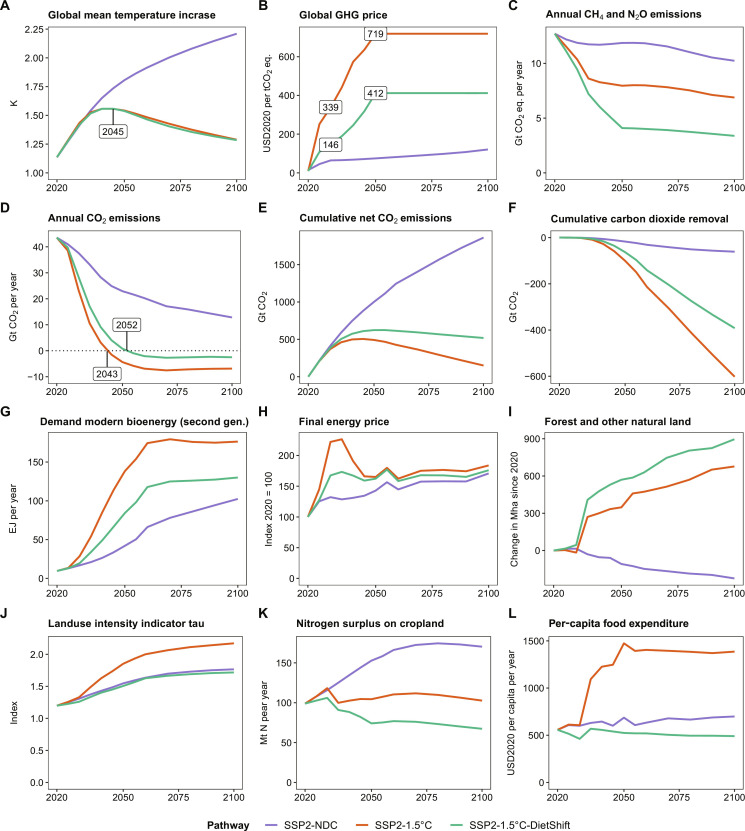
Key indicators for climate, energy, and land system development in the 21st century at global level from REMIND-MAgPIE. SSP2-NDC is shown as reference for a pathway that is not in line with the Paris Agreement. Peak global mean temperature increase (**A**) is identical in SSP2-1.5°C and SSP2-1.5°C-DietShift. The global GHG price (**B**) in SSP2-1.5°C-DietShift is 57% lower in 2030 and 43% lower in 2050 compared to SSP2-1.5°C. CH4 and N2O emissions (**C**) decrease considerably in SSP2-1.5°C-DietShift, which relaxes the tight constraint on the CO_2_ emission budget (**D** and **E**). Reliance on CDR (**F**) as well as demand for bioenergy (**G**) are lower in SSP2-1.5°C-DietShift, while final energy prices (**H**) increase less strongly. Forest and other natural land (**I**) increase stronger under SSP2-1.5°C-DietShift, while land use intensity (**J**) is lower and comparable to SSP2-NDC. Nitrogen surplus (**K**) decreases stronger in SSP2-1.5°C-DietShift and per-capita food expenditures for agricultural products (**L**) remain rather constant in the 21st century instead of a doubling in SSP2-1.5°C.

**Fig. 3.**
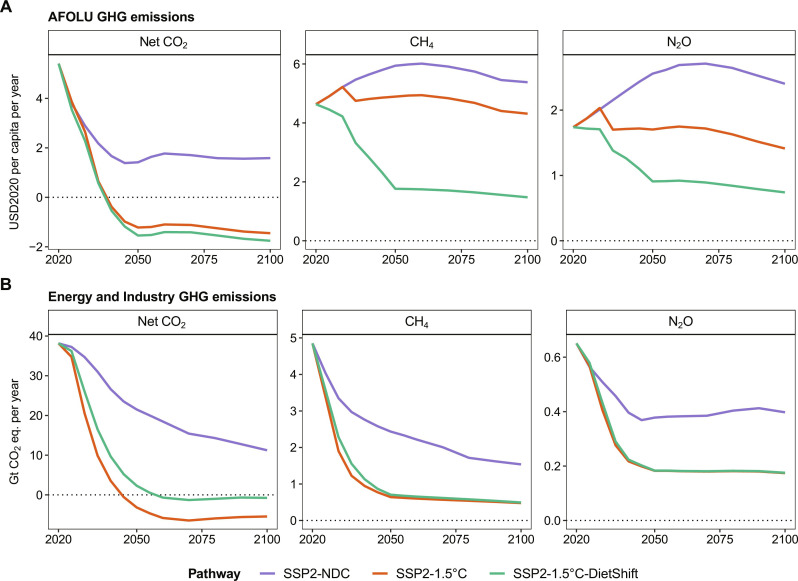
Sectoral GHG emissions at global level in the 21st century. (**A**) GHG emissions from AFOLU, which include net CO_2_ emissions from land-use change (deforestation and loss of other natural land) and regrowth of natural vegetation (re-/afforestation and land abandonment), CH_4_ emissions from enteric fermentation, animal waste management, rice cultivation and waste burning, and N_2_O emissions from agricultural soils, animal waste management and waste burning. (**B**) GHG emissions from energy and industry, which include net CO_2_ emissions from energy (including BECCS), industry, and other not land-based CDR options [direct air carbon capture and storage (DACCS) and synthetic fuels]; CH_4_ emissions from energy supply; extraction and waste; and N_2_O emission from energy supply, industry, transport, and waste. N_2_O and CH_4_ emissions have been converted into CO_2_ equivalents using IPCC AR6 GWP100 factors of 273 and 27, respectively.

**Fig. 4.**
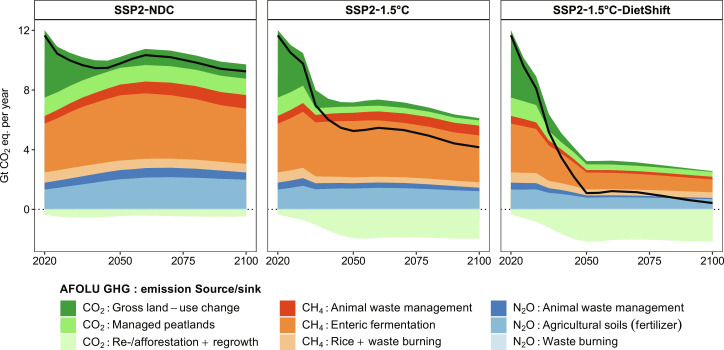
Global GHG emissions from AFOLU in the 21st century. GHGs (CO_2_, CH_4_, and N_2_O) are grouped by color and are further differentiated into subcategories of emission sources and sinks. N_2_O and CH_4_ emissions have been converted into CO_2_ equivalents using IPCC AR6 GWP100 factors of 273 and 27, respectively. Black solid lines show the sum (net effect) of all AFOLU GHG emission sources and carbon sinks (re-/afforestation + regrowth).

**Fig. 5.**
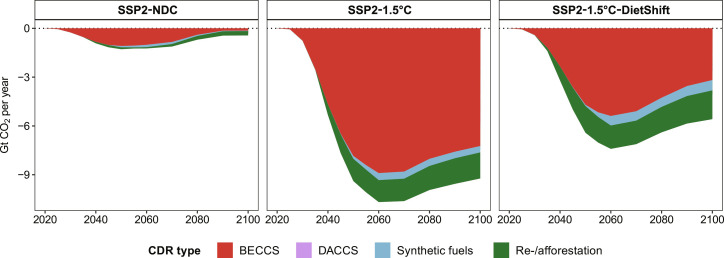
CDR at global level in the 21st century. CDR options include BECCS, DACCS, synthetic fuels with carbon capture and storage and re-/afforestation.

Global warming compared to pre-industrial times reaches 2.21°C in 2100 in the SSP2-NDC pathway. Therefore, current and promised national climate policies are insufficient to meet the Paris Agreement goal of limiting global mean temperature increase to 1.5°C above pre-industrial levels. The SSP2-1.5°C pathway with a peak carbon budget of 500 GtCO_2_ from 2020 onward shows that extending NDCs with comprehensive GHG emission pricing covering the energy as well as the land use system in all regions globally can limit global warming to a peak temperature increase of 1.56°C in 2045 (Fig. 2A). It is thus in line with the IPCC AR6 Working Group III (WG3) classification of 1.5°C pathways with no or low overshoot (*1*). The SSP2-1.5°C-DietShift pathway is designed to match the peak temperature increase of 1.56°C in 2045 of the SSP2-1.5°C pathway, which increases the 1.5°C compatible peak carbon budget from 500 to 625 GtCO_2_ under SSP2-1.5°C-DietShift (Table 1 and Materials and Methods). Therefore, both pathways can be considered 1.5°C compatible (IPCC AR6 WG3 category C1). By 2050, global GHG prices increase to 75, 719, and 412 USD2020 per metric ton CO_2_-eq in SSP2-NDC, SSP2-1.5°C, and SSP2-1.5°C-DietShift, respectively (Fig. 2B). Therefore, the mid-term 1.5°C compatible global GHG price for 2050 is 43% lower under dietary shifts (Table 2). Notably, the short-term 1.5°C compatible GHG price for 2030 is 57% lower under dietary shifts (339 USD2020 per metric ton CO_2_-eq in SSP2-1.5°C and 146 USD2020 per metric ton CO_2_-eq in SSP2-1.5°C-DietShift). The main reasons for this are lower CH_4_ and N_2_O emissions from agriculture under dietary shifts (Figs. 2C and 3A), which relax the tight constraint on CO_2_ emissions in the energy system (Figs. 2D and 3B) for the same climate outcome in terms of peak temperature increase in 2045 (Fig. 2A). It is important to highlight the special role of CH_4_ emission reductions for this result. Lower CH_4_ emissions from ruminant enteric fermentation (meat and milk products) account for the largest share of Agriculture, Forestry and Other Land Use (AFOLU) GHG emissions reductions under dietary shifts (Fig. 4). The Global Warming Potential (GWP100) of one unit of CH_4_ is 27 times higher compared to one unit of CO_2_ (*20*). Moreover, CH_4_ is a short-lived climate forcer with an average atmospheric lifetime of only 12 years compared to centuries for CO_2_ (*20*). Therefore, avoided CH_4_ emissions have short-term benefits for the climate system or, as assumed here, allows for more GHG emissions from other sectors for the same climate outcome. The delay of net-zero CO_2_ emissions from 2043 to 2052 (Fig. 2D) under dietary shifts increases the 1.5°C compatible carbon budget from 500 to 625 GtCO_2_ (Fig. 2E), while total cumulative CDR is about 39% lower in 2050 and 35% lower in 2100 (Fig. 2F). The main reason for this is less bioenergy with carbon capture and storage (BECCS; Fig. 5), which is also reflected in lower demand for modern second generation bioenergy under dietary shifts (Fig. 2G). Moreover, aggregated final energy prices more than double (increase by 122%) between 2020 and 2030 under SSP2-1.5°C. The relaxed constraint on CO_2_ emissions in SSP2-1.5°C-DietShift limits this short-term increase of final energy prices to 68%, which is still considerable. However, even in the SSP2-NDC case, energy prices rise by 32% due to the start of many NDCs between 2020 and 2030 (Fig. 2H). Particularly, final energy prices for solids and gases, which are harder to decarbonize, are lower under dietary shifts (fig. S7). Forest and other natural land declines by 224 Mha in SSP2-NDC and increases by 677 Mha in SSP2-1.5°C and 896 Mha in SSP2-1.5°C-DietShift between 2020 and 2100 (Fig. 2I). At the same time, dietary shifts by 2050 mitigate pressure on scarce land resources (Fig. 2J) and reduces nitrogen losses to the environment (Fig. 2K). In addition, dietary shifts keep food expenditures for agricultural products in a 1.5°C pathway at present-day levels (SSP2-1.5°C-DietShift) and thus avoid a doubling of food expenditures by 2050 as in the 1.5°C case without dietary shifts (Fig. 2L). Last, the 1.5°C pathway with dietary shifts shows economic welfare gains relative to the case without dietary shifts (Table 2) because lower costs for climate policy (reflected in lower GHG prices) in combination with a second-order effect on income (fig. S9) leave more income available for the consumption of goods. Consumption (cumulative over time and discounted at a 5% annual rate to 2020) in SSP2-1.5°C-DietShift is 2.8% higher in the period 2020–2050 and 2.1% higher in the period 2020–2100 compared to SSP2-1.5°C.

**Table 2.** Impact of dietary shifts on key economic indicators in 1.5°C compatible pathways. The first row shows cumulative discounted consumption (present value in 2020 based on a 5% discount rate) in SSP2-1.5°C-DietShift relative to SSP2-1.5°C for the periods 2020–2050 and 2020–2100, respectively. The second row shows the reduction of GHG prices in SSP2-1.5°C-DietShift relative to SSP2-1.5°C in 2050 and 2100, respectively.

	(2020)–2050	(2020)–2100
**Cumulative discounted welfare gain due to dietary shifts under 1.5°C**	2.8%	2.1%
**GHG price reduction due to dietary shifts under 1.5°C**	43%	43%

### Dietary shifts considerably reduce AFOLU GHG emissions

In SSP2-NDC, global net AFOLU GHG emissions slightly decline from 11.7 GtCO_2_-eq in 2020 to 9.8 GtCO_2_-eq in 2050 and 9.3 GtCO_2_-eq in 2100 (Fig. 4 and fig. S14). This decline is due to lower CO_2_ emissions from land-use change (NDCs on re-/afforestation and reduced deforestation), while CH_4_ and N_2_O emissions from agriculture increase mostly because of increasing demand for livestock products in low- and middle-income regions (fig. S5A). GHG emission pricing in the land system in SSP2-1.5°C reduces net global AFOLU GHG emissions to 5.3 GtCO_2_-eq in 2050 and 4.2 GtCO_2_-eq in 2100 (reduction of 46 and 55% compared to SSP2-NDC, respectively). Net CO_2_ emissions from land-use change are negative due to additional re-/afforestation in response to the CO_2_ price (Fig. 2I). In SSP2-1.5°C, reduction of non-CO_2_ emissions is mostly achieved through technical mitigation options at the farm level such as animal health monitoring and improved management of animal waste (Fig. 4). Dietary shifts toward less animal-based products on top of GHG emission pricing reduce net global AFOLU GHG emissions to 1.1 GtCO_2_-eq in 2050 and 0.4 GtCO_2_-eq in 2100 (reduction of 89 and 96% compared to SSP2-NDC, respectively). Thus, dietary shifts combined with GHG emission pricing (SSP2-1.5°C-DietShift) roughly double the reduction of global net AFOLU GHG emissions compared to emission pricing only (SSP2-1.5°C).

### Dietary shifts free up agricultural land

**Fig. 6.**
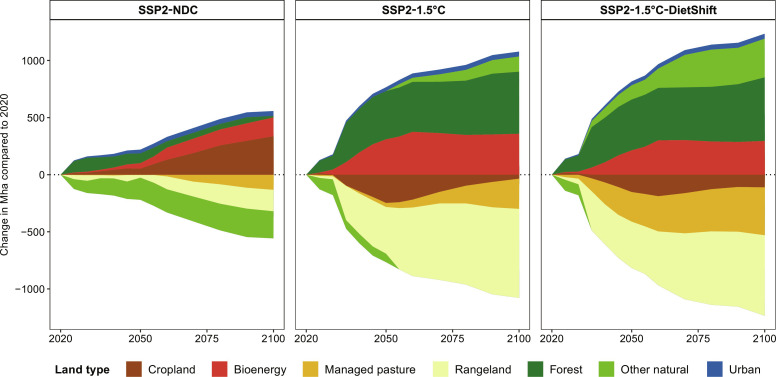
Land-use change of major land types at global level in the 21st century. Cropland includes food, non-food, and feed crops. Bioenergy includes second generation bioenergy (fast growing grasses and trees such as miscanthus and poplar). Grazing area is separated in managed pasture and rangeland. Forest includes primary forest, secondary forest, timber plantations, and re-/afforestation areas. Other natural land is a residual category, which includes among others nonforest ecosystems, deserts, and shrublands. Urban land includes built-up area.

Land dynamics in SSP2-NDC are shaped by cropland expansion mostly at the cost of other natural land, managed pasture, and rangeland (Fig. 6 and fig. S15). The main driver for cropland expansion is increasing total demand for crops and livestock products in consequence of rising global population and shifts toward more livestock products (figs. S5 and S10). However, when combined with GHG emission pricing in the land system as in SSP2-1.5°C, the same food demand trajectory results in much less cropland expansion and loss of other natural land. One reason for these dynamics is the price on CO_2_ emissions from land-use change, which makes cropland expansion into forest and other natural land costly. In consequence, there is a shift from land expansion to land-use intensification (Fig. 2J), which increases crop yields. The cost for this yield-increasing technological change is reflected in considerably higher food expenditures for agricultural products (Fig. 2L). Moreover, land-based CDR via BECCS and re-/afforestation plays a central role in SSP2-1.5°C (Fig. 5). A large part of the area required for bioenergy and re-/afforestation is sourced from the reduction of rangelands, which are often used extensively. At the same time, land-based CDR is an additional driver for land-use intensification. Dietary shifts in SSP2-1.5°C-DietShift strongly alter the land use dynamics compared to SSP2-1.5°C (Fig. 6). Even more rangelands and managed pastures are re-/afforested or abandoned, resulting in natural regrowth. At the same time, land-use intensity (Fig. 2J and fig. S16) is comparable to the case without GHG emission pricing (SSP2-NDC). Therefore, dietary shifts in general reduce pressure on scarce land resources, which is reflected in (i) a reduction of agricultural land and (ii) less intensification on the remaining agricultural land. Less intensification is also reflected in lower nitrogen losses and rather constant instead of more than doubling food expenditures for agricultural products throughout the 21st century (Fig. 2L).

## DISCUSSION

Our results show that dietary shifts toward a flexitarian healthy diet could increase the economic and physical feasibility of 1.5°C pathways, indicated by 43% lower GHG prices and 39% less CDR in 2050 compared to a case without dietary shifts. In line with previous studies, dietary shifts toward EAT-Lancet recommendations and calorie intake for a healthy BMI are exogenous assumptions in our modeling framework (*3*, *11*, *13*). Transaction costs for policymaking, implementation, and monitoring are not accounted for. Therefore, it is beyond the scope of this study to provide answers on how dietary shifts could be achieved. Nevertheless, our study provides additional arguments for transitioning to healthy diets and thus for research on policy instruments that aim to alter dietary patterns. In the following, we discuss price-based interventions and instruments that target food environments or consumer preferences, all in relation to our study.

The development of an actionable strategy for dietary shifts relies on the profound understanding of the political economy context and the identification of entry points and barriers for change (*17*, *21*). Putting a tax on meat consumption as measure to achieve dietary shifts has been controversially discussed in the literature (*13*, *22*, *23*). In general, food demand is rather inelastic to price changes and methodological limitations in the currently available literature estimates systematically overestimate food price elasticities (*13*). Moreover, even the available estimates are very inelastic to price changes and would require enormous tax rates—beyond the domain at which measured price elasticities can be applied with confidence—to achieve dietary shifts in line with healthy diets (*23*). Therefore, similar to (*13*), the cost-efficient GHG emission prices assumed in our study, which are central for reducing AFOLU GHG emissions on the supply side, would not result in food price changes high enough for triggering a transformative impact on dietary patterns. On the other hand, own-price elasticities for meat are higher than those for cereals, and ruminant meat has the highest own-price elasticity within the meat category followed by pork and poultry (*24*–*28*). Therefore, a high tax on beef and lamb only, which have the highest GHG emissions per unit of product across all animal products (*29*), could potentially contribute to CH_4_ emission reductions, which are central for the outcome of this study, while still allowing consumers to switch to pork or chicken. Even more promising are policy instruments that, rather than changing the monetary incentive structure, change food environments (e.g., food provision in canteens and food distribution schemes) or target the preferences of consumers as such (e.g., advertisement bans, education, or nutrition counseling) (*13*, *17*). A literature review analyzing 99 studies on food system interventions indicates that informing consumers about environmental, health, and animal welfare aspects of dietary patterns can effectively reduce meat consumption (*30*). The main result of our study, namely, that shifts toward healthy diets increase the feasibility of 1.5°C pathways, could therefore feed into policy instruments that directly target consumer preferences via fact-based messages. However, no estimates yet exist about the magnitude of dietary shifts that could be achieved by these interventions. Our study therefore reemphasizes the need for more research on policy instruments that target food environments and consumer preferences. In addition, decision-making in food policy is often dispersed across different institutions and ministries, which hinders the implementation of coherent policies in support of healthy diets (*31*). Moreover, there are concerns about job and income losses in upper-middle and high-income countries when less livestock products are consumed (*17*). Therefore, social inclusion and compensation schemes are central for a just transition to healthy diets (*17*, *21*, *32*). These challenges highlight the considerable difficulties of implementing healthy diets at global scale in the near term as assumed in the SSP2-1.5°C-DietShift pathway.

In upper-middle-income to high-income countries, the cost of healthy diets compared to the cost of current diets are estimated up to 22 to 34% cheaper (*33*). However, in low-income to lower-middle-income countries, which currently include a population of more than 3 billion people, healthy diets are at least 18 to 29% more expensive than current diets (*33*). The adoption of healthy diets is largely constrained by income. Shifts toward healthy diets in these countries are therefore only achievable if combined, e.g., with transfer payments or (revised) food distribution schemes. In this study, however, we did not investigate how these transfer schemes could be designed, which is a highly relevant research topic for the implementation of healthy diets. In the context of most low-income countries, vegetarian and vegan healthy diets would remain more affordable than the flexitarian healthy diet assumed in our scenarios (*33*). Yet, a more complete cost accounting that includes the diet-related costs of climate change and health care would make healthy diets the least costly option in most countries in the future (*33*).

Our study highlights the interdependence of energy, food, and land systems for the feasibility of the 1.5°C target. Previous studies identified numerous benefits of healthy diets on human health, terrestrial ecosystems, and AFOLU GHG emissions (*3*–*5*, *11*, *34*). Our results show that dietary shifts do not only reduce impacts from food production within the land system (e.g., land use and nitrogen losses) but also relax the 1.5°C compatible peak carbon budget by 125 GtCO_2_ via lower non-CO_2_ emissions from agriculture. In particular, CH_4_ emissions from ruminant enteric fermentation are lower under dietary shifts, which has short-term effects on the climate system because CH_4_ is a short-lived (12 years) but high-impact GHG (GWP100 factor of 27). Therefore, the 1.5°C pathway with dietary shifts (SSP2-1.5°C-DietShift) achieves the same climate outcome as SSP2-1.5°C (peak temperature increase of 1.56°C in 2045) but with less CDR and less stringent CO_2_ emission reductions in the energy system, which cuts GHG emission prices by 57% in 2030 and 43% in 2050. Moreover, the 1.5°C pathway with dietary shifts shows economic welfare gains and lower energy prices compared to the 1.5°C pathway without dietary shifts. For food expenditures, there are two counteracting effects. GHG emission pricing increases food expenditures for agricultural products (SSP2-1.5°C), while dietary shifts reduce food expenditures due to lower demand for resource-intensive animal-based products. In combination (SSP2-1.5°C-DietShift), dietary shifts largely offset food expenditure increases caused by 1.5°C compatible climate policies at global scale. However, potential rebound effects in food choices due to lower prices on agricultural markets (e.g., emission-intensive livestock products) are not accounted for in this study. These system-wide effects of dietary shifts as single measure under SSP2 middle-of-the-road assumptions on the feasibility of 1.5°C have not been analyzed previously but are of high relevance for policymaking.

Limiting warming to 1.5°C would considerably reduce the risk of crossing multiple climate tipping points compared to 2°C or above warming (*35*). However, even 1.5°C is not safe as five tipping elements might cross their physical thresholds at or before 1.5°C warming (*35*). The carbon budget of 500 GtCO_2_ applied in our study in the SSP2-1.5°C pathway is equal to the central estimate for the remaining carbon budget for limiting global warming to 1.5°C with a 50% likelihood (IPCC AR6 WG1 Table 5.8) (*36*). However, the remaining carbon budget is subject to large uncertainties, with the non-CO_2_ warming contribution as a major determinant of its size. Our results show that the reduction of non-CO_2_ emissions under SSP2-1.5°C-DietShift increases the available carbon budget, for the same peak warming as in SSP2-1.5°C, by 125 to 625 GtCO_2_. Vice versa, keeping the carbon budget at 500 GtCO_2_ in combination with the non-CO_2_ emission reductions from dietary shifts would increase the likelihood of limiting warming to 1.5°C. The 125 GtCO_2_ difference in allowable CO_2_ budgets derived in our study compares to the difference of 100 GtCO_2_ between the CO_2_ budgets for 50 and 67% likelihood of keeping warming below 1.5°C, as assessed by the IPCC AR6 WG1 (*36*). These results indicate that dietary shifts could make a difference for limiting global warming to below 1.5°C, which calls for globally concerted efforts to support the transition toward sustainable healthy diets.

## MATERIALS AND METHODS

### IAM REMIND-MAgPIE

The IAM REMIND-MAgPIE combines the energy-economy model REMIND (regional model of investments and development) with the food and land-use model MAgPIE (model of agricultural production and its impact on the environment) (*37*). Both models are mathematical optimization models with global coverage and identical regional setup. Moreover, key scenario assumptions of the SSP framework such as population and income are harmonized between the two models. In addition, REMIND endogenously accounts for second-order effects of climate policy on income. To reach an equilibrium, both models are iteratively run five times. After each run, REMIND provides information on GHG prices and second generation bioenergy demand to MAgPIE. Within MAgPIE, GHG prices are applied to CO_2_ emissions from land-use change and drained peatlands, and non-CO_2_ emissions from agriculture, while second generation bioenergy demand is added on top of agricultural demand. In addition, the CO_2_ price serves as economic incentive for re-/afforestation and rewetting of drained peatlands. The resulting GHG emissions and removals from the land system and the price for bioenergy are reported back from MAgPIE to REMIND and are considered in the next iteration. For modeling transformation pathways in line with the Paris Agreement, a peak carbon budget for the remaining cumulative CO_2_ emissions until net-zero annual CO_2_ emissions must be reached is imposed. CO_2_ emissions from all sectors of the economy, including the energy and land system, count toward the carbon budget. The combined effects of CO_2_ emissions and other major GHGs on radiative forcing and global mean temperature increase are derived by the simple climate model MAGICC6 (*38*). Both REMIND and MAgPIE express prices not in nominal but real terms, i.e., they are adjusted for inflation and given in USD prices for 2020. The IAM REMIND-MAgPIE has been used to simulate various mitigation pathways (*39*), two of which have been selected and highlighted as Illustrative Mitigation Pathways [IMP-SP (*3*) and IMP-Ren (*40*)] by the IPCC AR6 WG3 (*1*).

REMIND integrates a Ramsey-type growth model of the economy with an engineering-based energy system model (*40*, *41*). The two models link the energy demand generated by major sectors such as transport, industry, and buildings to the economic activity. The cost of energy use is considered by the economic core. The transformation of the energy sector is limited by factors such as inertia, path dependencies, and learning curves and adjustment costs associated with adopting new technologies. The emissions of major GHGs are linked to primary energy sources.

MAgPIE integrates a regional food demand model with a spatially explicit land-use allocation model (*42*, *43*). Regional food energy demand is defined on the basis of regional diets and 10 food energy categories for a given population. Future trends in food demand are derived from a cross-country regression analysis based on future scenarios on gross domestic product (GDP) and population growth. The goal function of MAgPIE is to fulfil a given demand of food, feed, and bioenergy at least cost under a set of economic and biophysical constraints including production costs, self-sufficiency ratios, land and water availability, and potential crop yields [from the Lund-Potsdam-Jena managed Land (LPJmL) model] (*44*–*47*). Crop yield increases due to technological change are modeled endogenously based on regionally different investment-yield ratios and interest rates (*48*). Hence, the model simultaneously optimizes the rate of yield-increasing technological change and cropland expansion, which is especially relevant for long-term projections. MAgPIE accounts for CO_2_ emissions from land-use change (e.g., deforestation) and for carbon uptake due to re-/afforestation and land restoration; all based on carbon stock changes (*11*, *12*). N_2_O emissions from agricultural soils (fertilizer application) and animal waste management are estimated on the basis of nitrogen budgets for croplands, pastures, and the livestock sector (*49*, *50*). CH_4_ emissions from agriculture include emissions from enteric fermentation, animal waste management and rice cultivation, which are estimated based on feed demand, manure, and rice cultivation area, respectively (*49*, *51*). CO_2_, CH_4_, and N_2_O emissions from managed peatlands (drained and rewetted) are calculated on the basis of IPCC wetland GHG emission factors (*52*).

### Scenario setup

All scenarios follow middle-of-the-road SSP2 assumptions with respect to population, income, diets and other drivers for land and energy system development (*53*, *54*). SSP2-NDC includes NDCs in the land and energy system. In the energy system, NDCs are represented by regionally fragmented GHG prices (*55*). In the land system, NDCs for re-/afforestation and reduced deforestation are explicitly modeled and remain constant after 2030. Climate change impact on crop yields, carbon densities, and water availability consistent with RCP 4.5 (Representative Concentration Pathway with a radiative forcing of 4.5 W/m^2^ in the year 2100) has been derived by LPJmL.

SSP2-1.5°C is a 1.5°C compatible transformation pathway. On top of NDCs, stringent emission reductions are imposed by a peak carbon budget of 500 GtCO_2_ from 2020 onward for combined land and energy system CO_2_ emissions. According to the IPCC AR6 WG1, a carbon budget of 500 GtCO_2_ from 2020 onward has a 50% chance of keeping global warming below 1.5°C (*36*). From 2035 onward, GHG prices from the energy system are also applied in the land system on AFOLU GHG emissions, which (i) increases the cost for conversion of forest and other ecosystems and (ii) activates technical mitigation options for the reduction of non-CO_2_ emissions. Conceptually, the pricing of AFOLU GHG emissions and technical abatement are identical to Humpenöder *et al.* (*11*), while the underlying marginal abatement cost curves have been updated for this study (*56*). Climate change impact on crop yields, carbon densities, and water availability consistent with RCP 1.9 (Representative Concentration Pathway with a radiative forcing of 1.9 W/m^2^ in the year 2100) has been derived by LPJmL.

The setup of SSP2-1.5°C-DietShift is identical to SSP2-1.5°C with two exceptions: (i) dietary shifts toward the EAT-Lancet Planetary Health Diet (flexitarian diet) and per-capita calorie intake for a healthy BMI by 2050 and (ii) a higher peak carbon budget of 625 GtCO_2_ from 2020 onward until the year of net-zero CO_2_ emissions. It is worth noting that the EAT-Lancet Planetary Health Diet is not based on the concept of Planetary Boundaries. Rather, the diet is designed on the basis of the findings of health research, with the goal of achieving optimal health outcomes for humans (*2*, *34*). Per-capita calorie intake in SSP2-1.5°C-DietShift does not converge to the identical value in 2050 for all regions but to regionally different estimates for calorie intake that is consistent with a healthy BMI, depending on the demographic structure, physical activity, and body height. Dietary shifts considerably reduce AFOLU GHG emissions, which in combination with a peak carbon budget of 500 GtCO_2_ would result in a lower peak temperature increase compared to SSP2-1.5°C. To maintain comparability of the two pathways regarding climate outcome, we determined the carbon budget for SSP2-1.5°C-DietShift, such that the peak temperature increase in 2045 is identical to SSP2-1.5°C*.* The implementation of per-capita dietary shifts are identical to Humpenöder *et al.* (*11*).

## Supplementary Materials

### This PDF file includes:

Figs. S1 to S16 Table S1 References
