# Supplementary material for: Food matters: Dietary shifts increase the feasibility of 1.5°C pathways in line with the Paris Agreement
Source: Sci Adv. 2024 Mar 27;10(13):eadj3832. doi: 10.1126/sciadv.adj3832 (PMC10971398; doi:10.1126/sciadv.adj3832)
Supplement: Supplementary file 1 — Figs. S1 to S16 Table S1 References [file sciadv.adj3832_sm.pdf]

Science Advances
AAAS

Supplementary Materials for

Food matters: Dietary shifts increase the feasibility of 1.5°C pathways in line with the Paris Agreement

Florian Humpenöder *et al.*

Corresponding author: Florian Humpenöder, humpenoeder@pik-potsdam.de

Sci. Adv. **10**, eadj3832 (2024)
DOI: 10.1126/sciadv.adj3832

This PDF file includes:

Figs. S1 to S16
Table S1
References

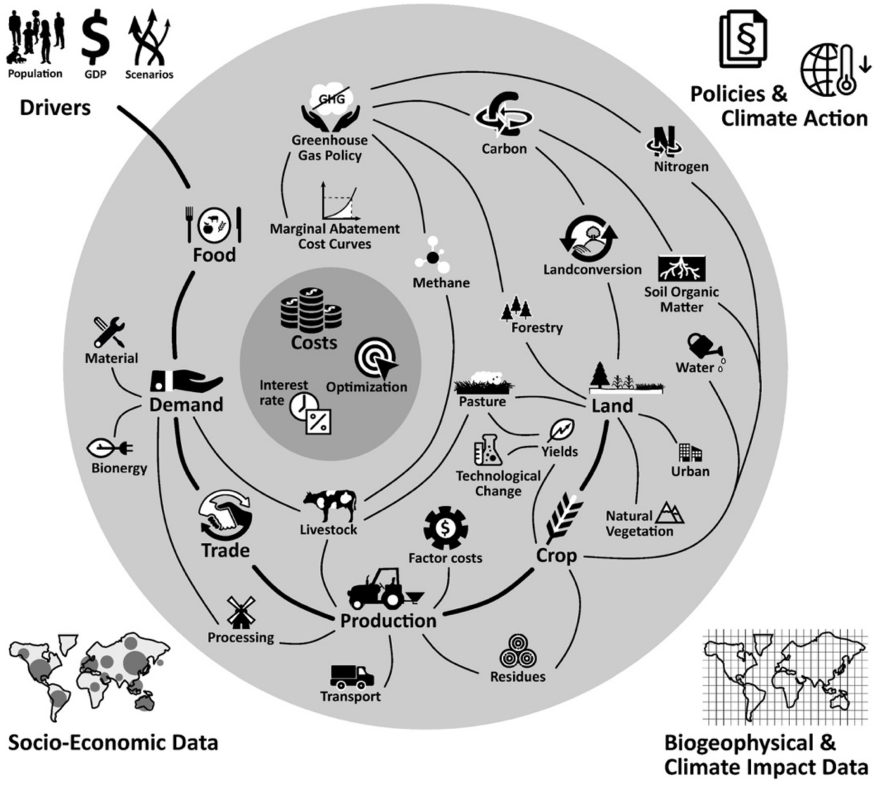

*Fig. S1: **MAgPIE 4 framework.** Simplified modular structure and module interactions. Reproduced from (42) under CC-BY-4.0 license.*

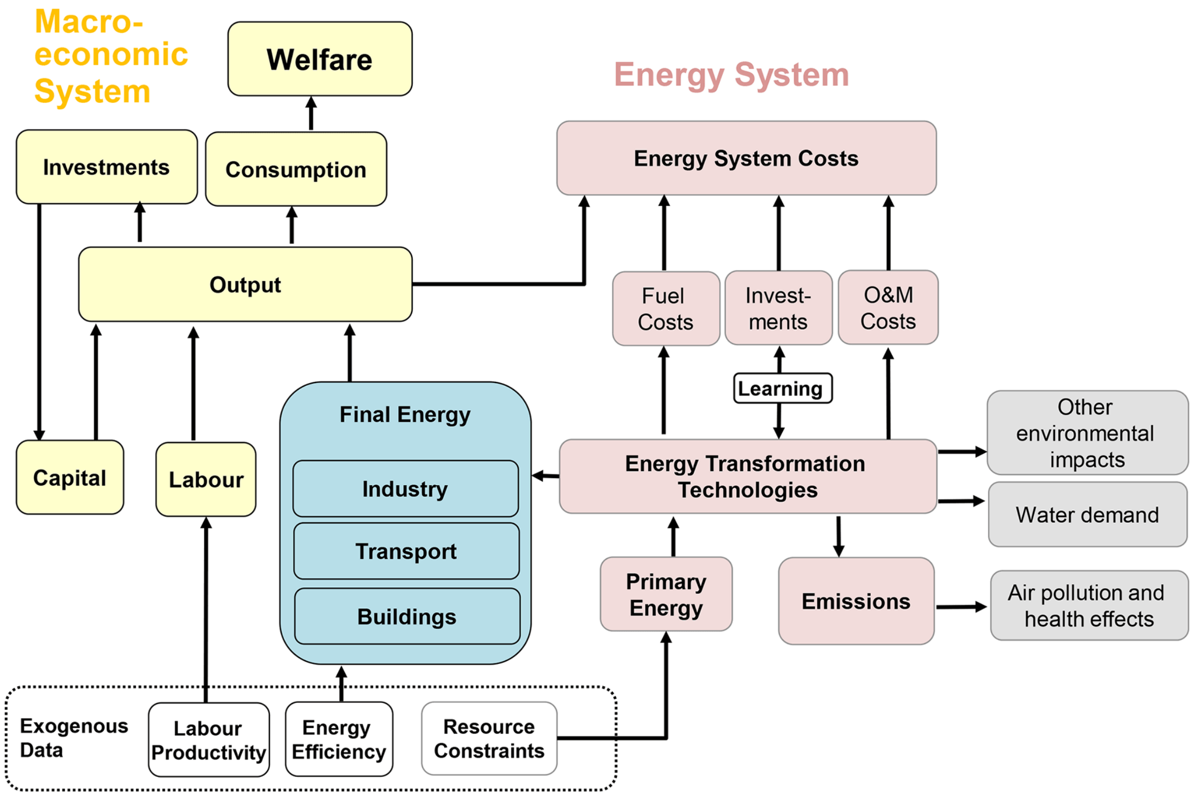

*Fig. S2: **Structure of REMIND.** Reproduced from (55) under CC-BY-4.0 license.*

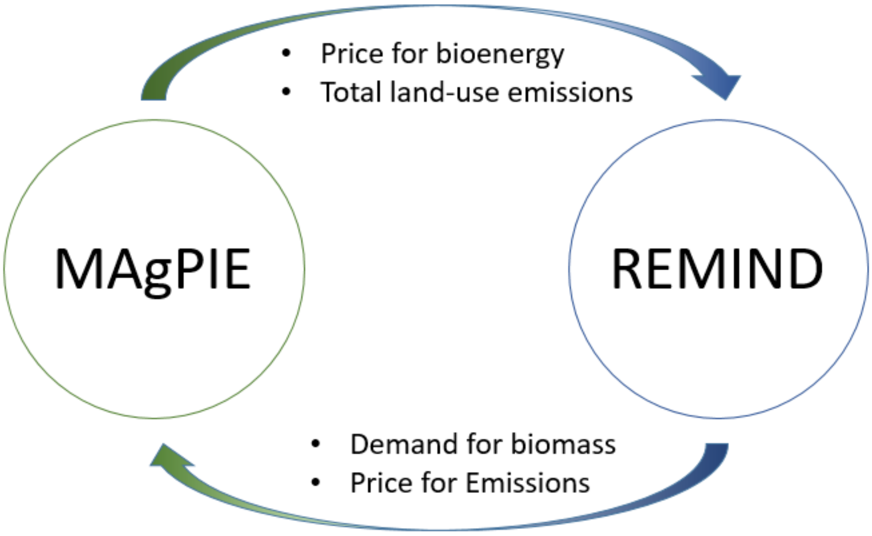

*Fig. S3: **Coupling of REMIND and MAgPIE.** Reproduced from the tutorial for running REMIND and MAgPIE in (41) under GNU AGPL-3.0 license.*

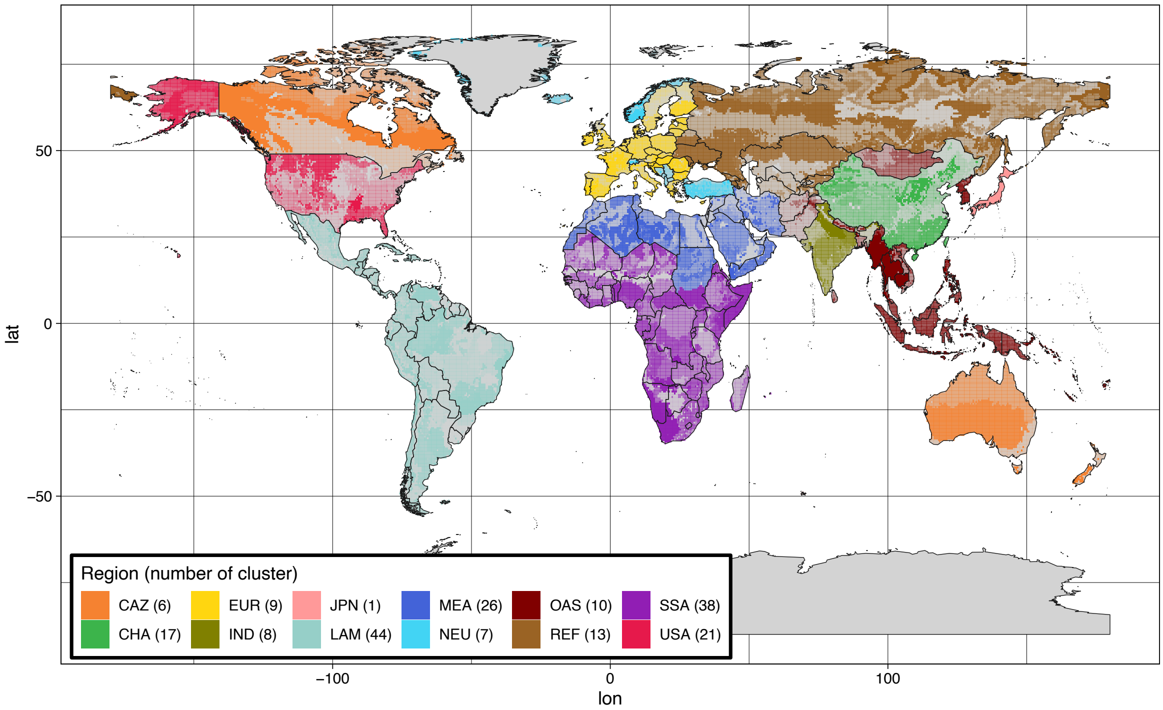

*Fig. S4: **Map of REMIND-MAgPIE regions and associated spatial simulation units.** Regional definitions: CAZ (Canada, Australia, and New Zealand; CHA (China); EUR (European Union); IND (India); JPN (Japan); LAM (Latin America); MEA (Middle East and north Africa); NEU (non-EU member states); OAS (other Asia); REF (reforming countries); SSA (Sub-Saharan Africa); USA (United States). The country map is based on the object " wrld_simpl" in the R package "maptools".*

Name of region	Income group	REMIND-MAgPIE regions
HIR	high-income	Canada, Australia, New Zealand (CAZ), European Union (EUR), United States (USA), Japan (JPN)
MIR	middle-income	Latin America (LAM), China (CHA), other Asia (OAS), non-EU member states (NEU), Middle East and North Africa (MEA), reforming countries (REF)
LIR	low-income	India (IND), Sub-Saharan Africa (SSA)

Table S1: ***Regional mapping.*** *Definition of aggregated regions used in this study, income group and the corresponding* MAgPIE regions (see *Fig. S4 for a map of REMIND-MAgPIE regions).*

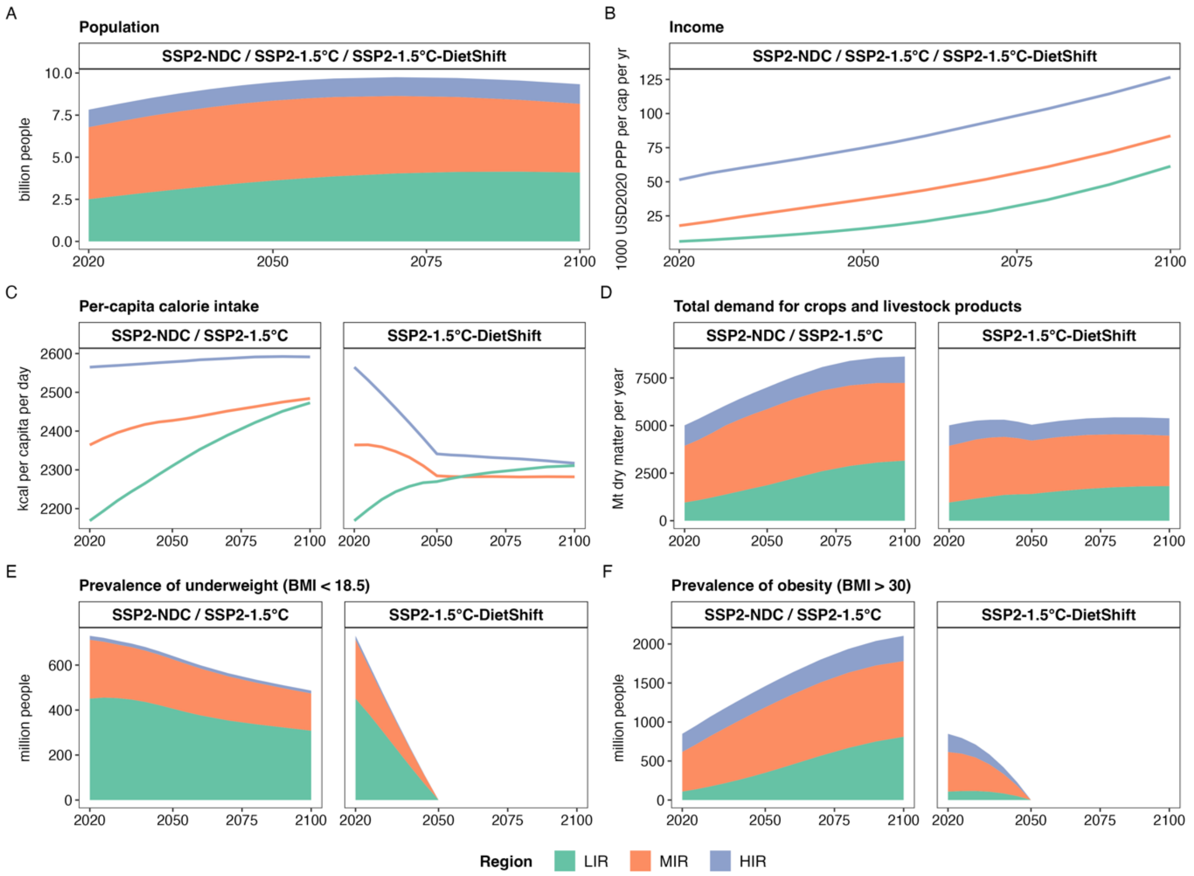

Fig. S5: ***Key socio-economic drivers and assumptions for future food demand in REMIND-MAgPIE.*** *Data is shown for three pathways (SSP2-NDC, SSP2-1.5°C and SSP2-1.5°C-DietShift) and has been aggregated to low-, middle and high-income regions (LIR, MIR and HIR; see Table S1 for mapping of regions). Population (A) and per-capita income (B) used for food demand calculations follow SSP2 middle-of-the-road trajectories. Per-capita calorie intake (C) follows SSP2 assumptions in SSP2-NDC / SSP2-1.5°C and shifts towards intake levels that correspond with a healthy body mass index (BMI) in SSP2-1.5°C-DietShift. Total global demand for crops and livestock products (for food, feed and other purposes) (D) increases in SSP2-NDC / SSP2-1.5°C and remains rather constant in SSP2-1.5°C-DietShift. Prevalence of underweight (E) slightly declines in SSP2-NDC / SSP2-1.5°C and is eliminated by 2050 in SSP2-1.5°C-DietShift. Prevalence of obesity (F) more than doubles in SSP2-NDC / SSP2-1.5°C and vanishes by 2050 in SSP2-1.5°C-DietShift.*

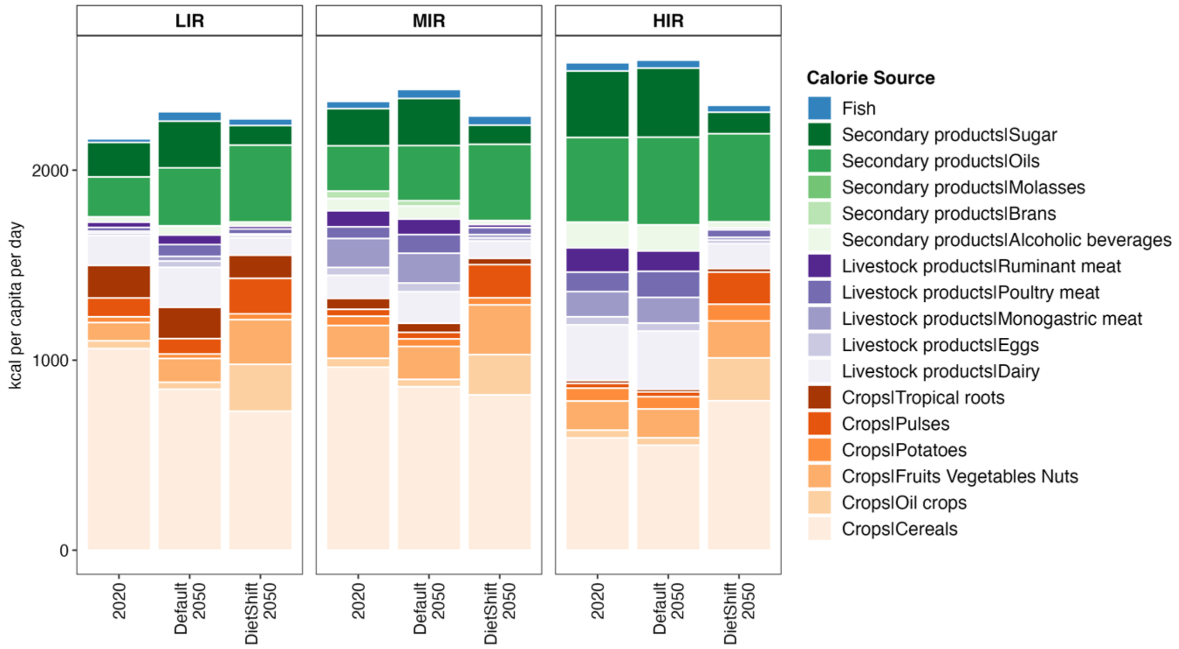

*Fig. S6: **Per-capita calorie intake and dietary composition.** Data is shown for 2020, Default 2050 (SSP2-NDC / SSP2-1.5°C) and DietShift 2050 (SSP2-1.5°C-DietShift). Data has been aggregated to low-, middle and high-income regions (LIR, MIR and HIR; see Table S1 for mapping of regions). In the SSP2-1.5°C-DietShift pathway, all regions shift towards EAT-Lancet recommendations (planetary health diet) by 2050.*

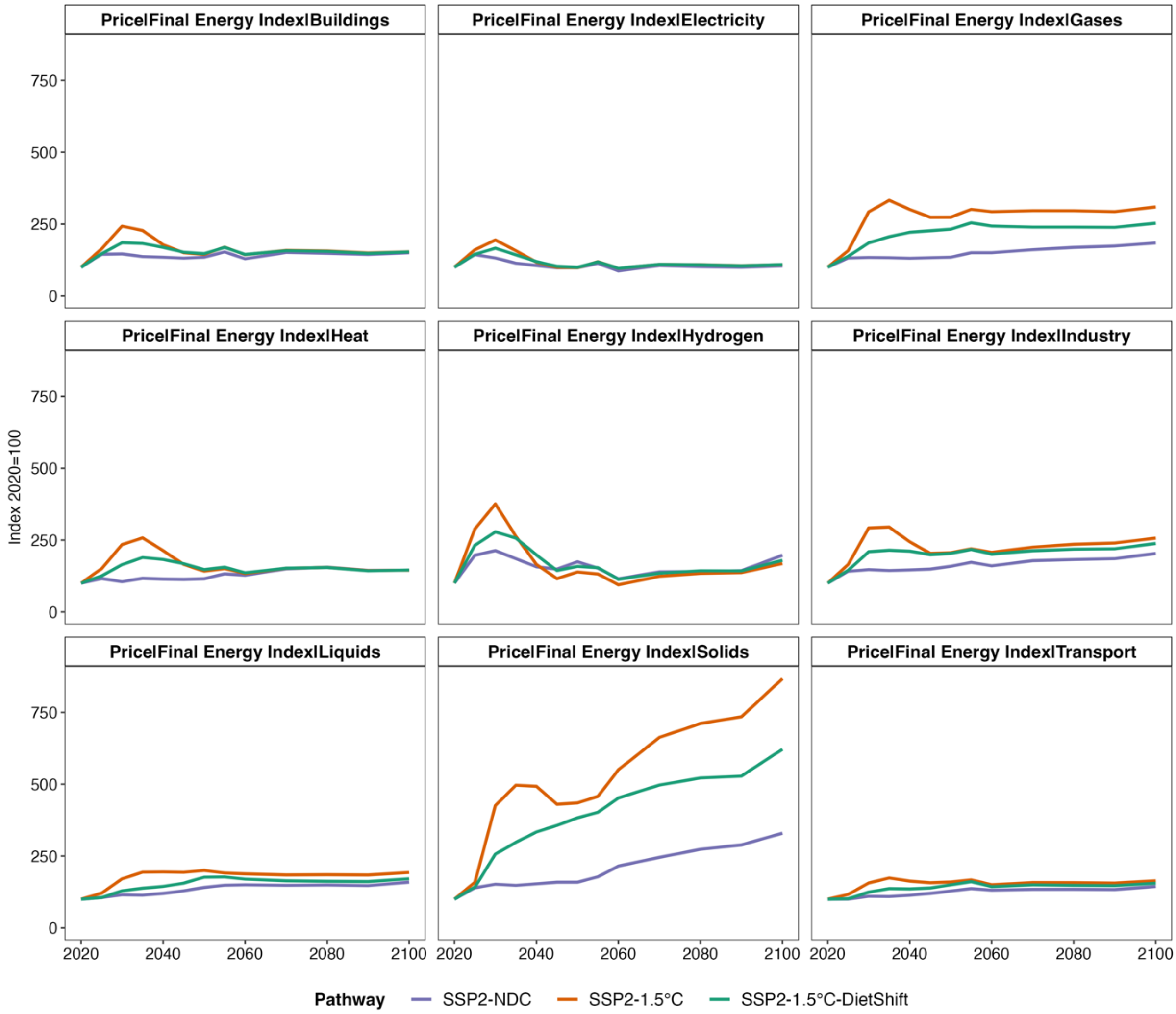

Fig. S7: ***Final energy price indices at global level in the 21st century.***

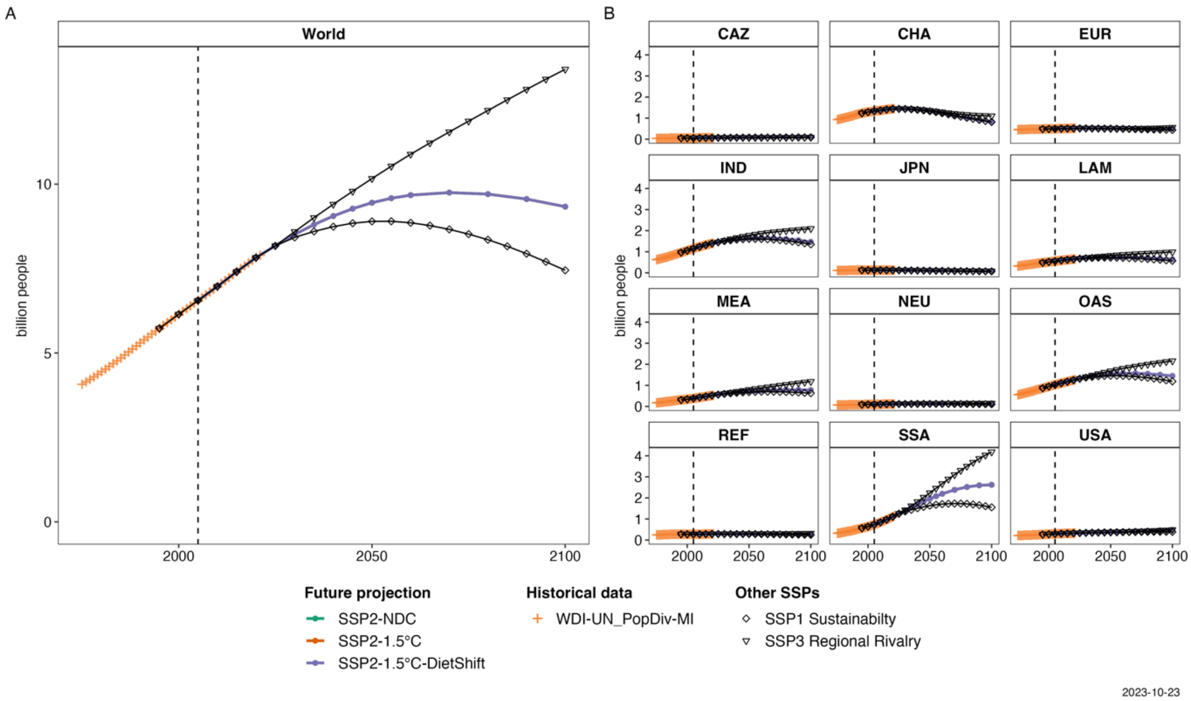

*Fig. S8: **Population.** A) shows global data. B) shows regional data. Population in this study follows SSP2 assumptions (same for all pathways). SSP1 and SSP3 are shown for context only. Population projections are based on KC and Lutz (57). Historical data for comparison from World Bank World Development Indicators (WDI) (58). The historical data has been processed using the pik-piam/mrvalidation R package (59).*

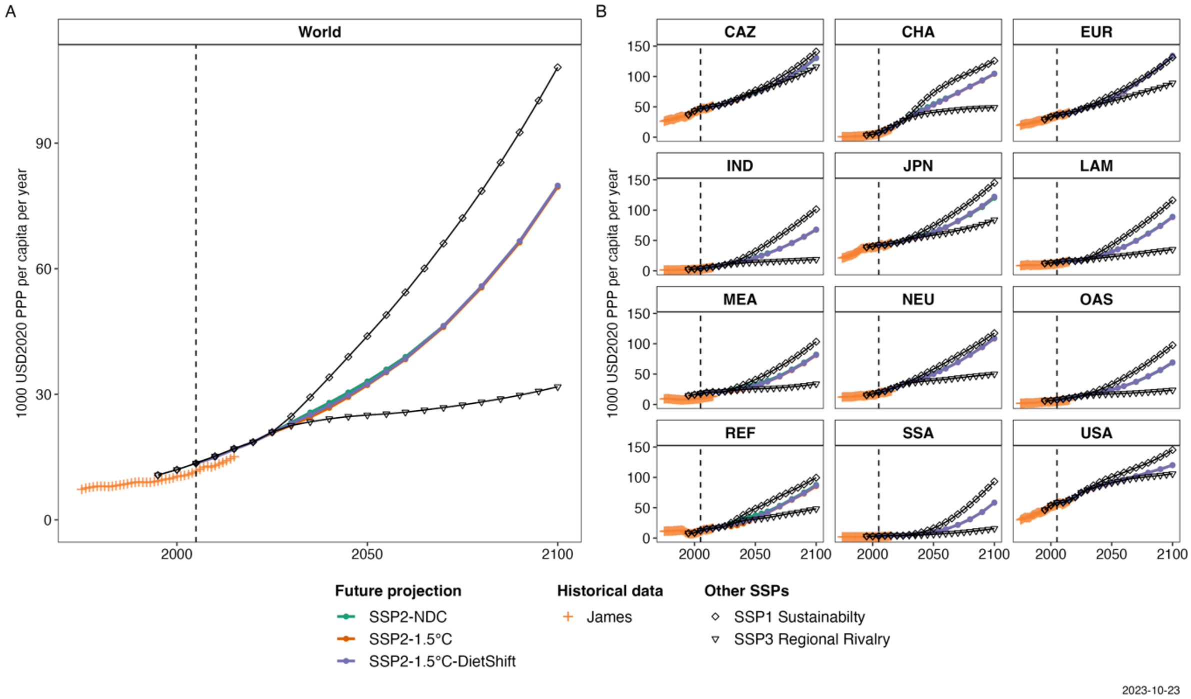

*Fig. S9: **Income.** A) shows global data. B) shows regional data. Income in this study is calibrated to SSP2 assumptions in all pathways. Income is very similar but not identical in all pathways because of model-endogenous second-order effects of climate policy on income. SSP1 and SSP3 are shown for context only. Income projections are based on Dellink et al (60). Historical data for comparison from James et al (61).The historical data has been processed using the pik-piam/mrvalidation R package (59).*

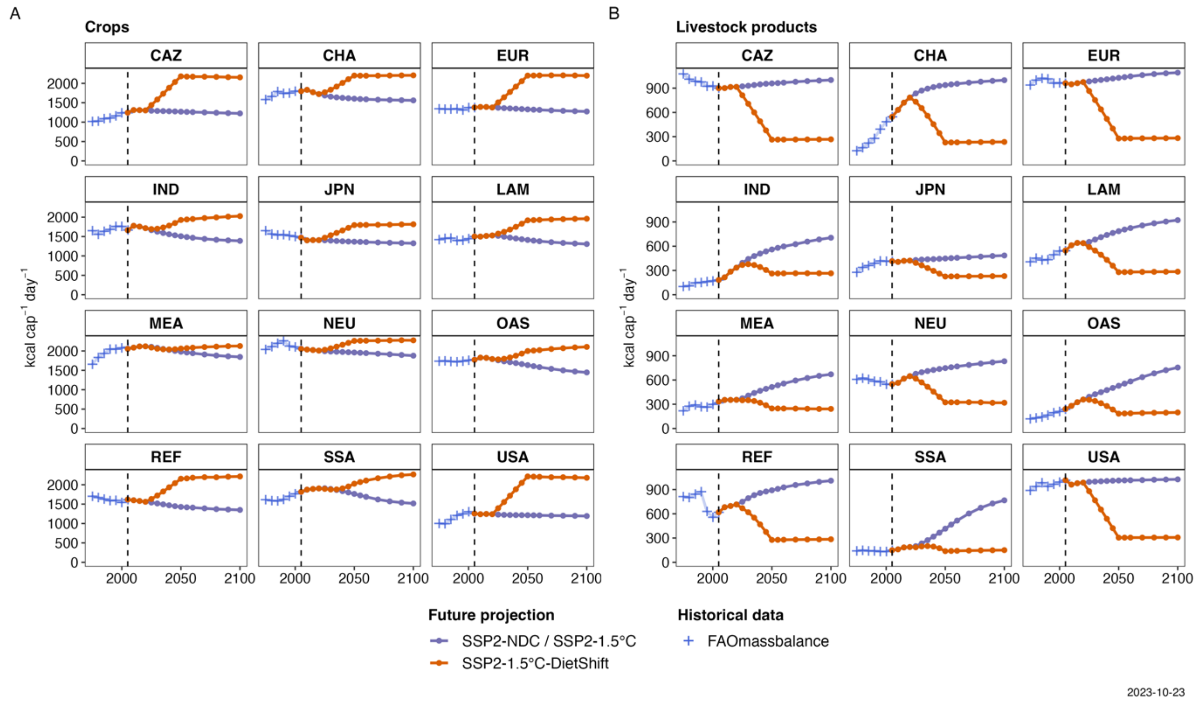

Fig. S10: ***Per-capita calorie supply.*** *Calorie supply is the sum of calorie intake and food waste. Data is shown at regional level. A) shows crops. B) shows livestock products. Historical data from FAO (62). The historical data has been processed using the pik-piam/mrvalidation R package .*

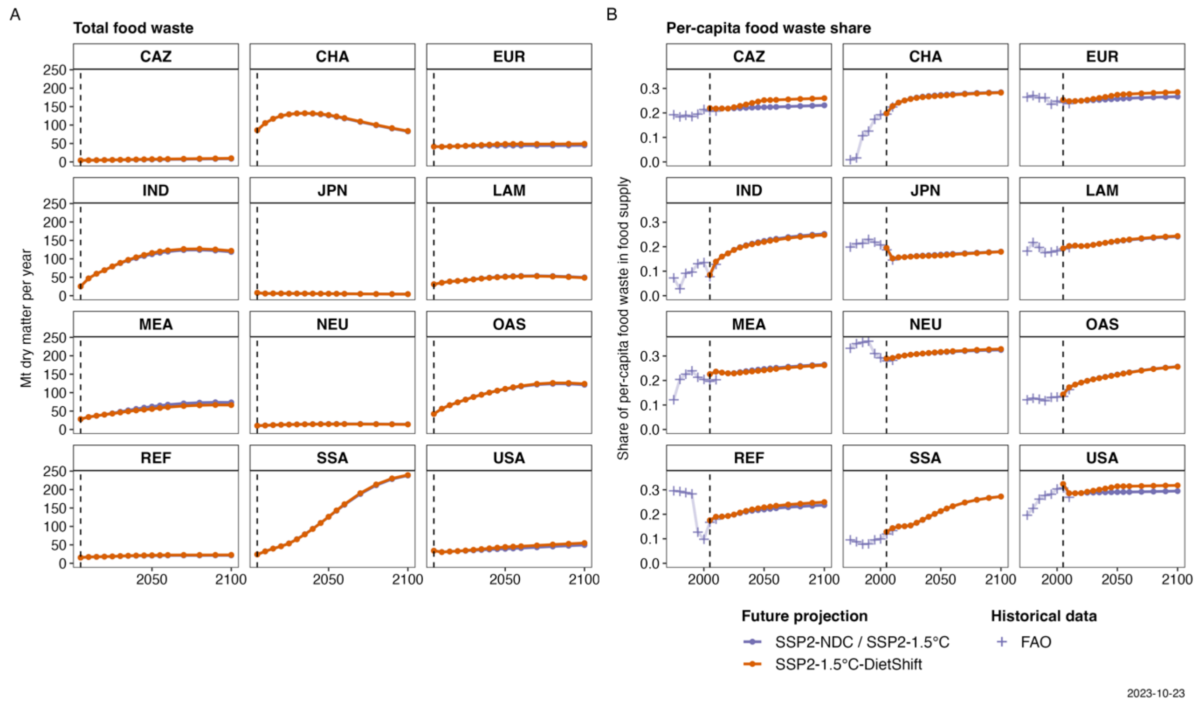

Fig. S11: ***Food waste.*** *A) shows total food waste in million ton dry matter per year. B) shows per-capita food waste as share of per-capita calorie supply. Data is shown at regional level. Historical data from FAO (62). The historical data has been processed using the pik-piam/mrvalidation R package (59).*

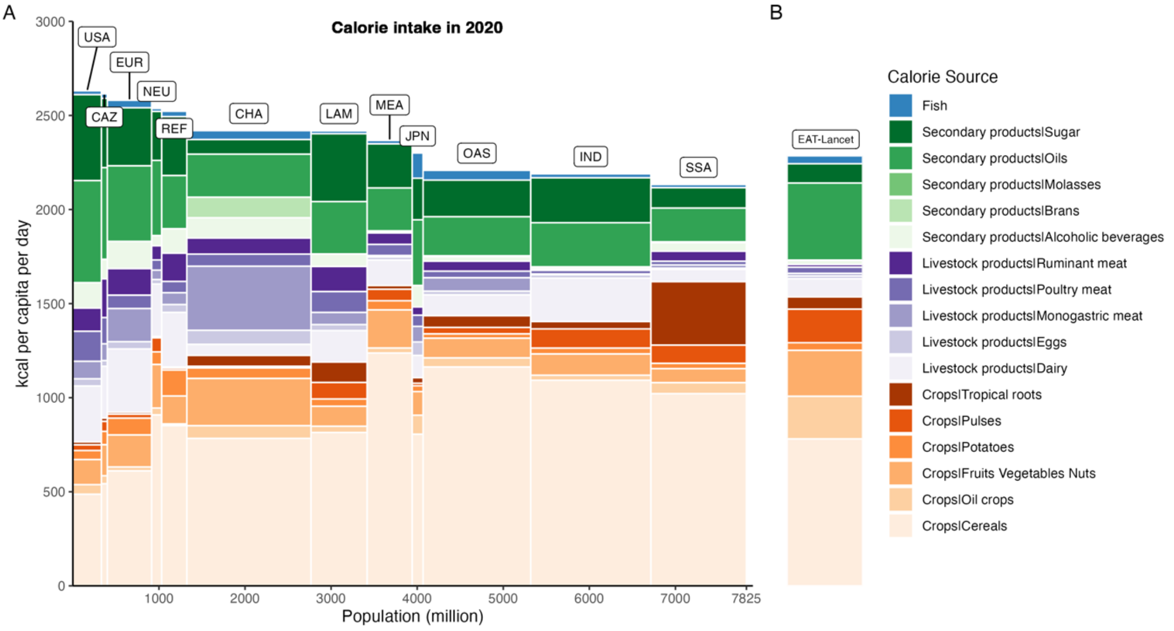

*Fig. S12: **Per-capita calorie intake, dietary composition and population.** A) shows regional data for 2020. The height of each rectangle shows per-capita kcal intake, the width shows the population of the region, so that the area of the rectangles refers to the total calorie intake for each region. B) shows EAT-Lancet recommendations (planetary health diet), aggregated to global level. In the SSP2-1.5°C-DietShift pathway, all regions shift towards EAT-Lancet recommendations by 2050.*

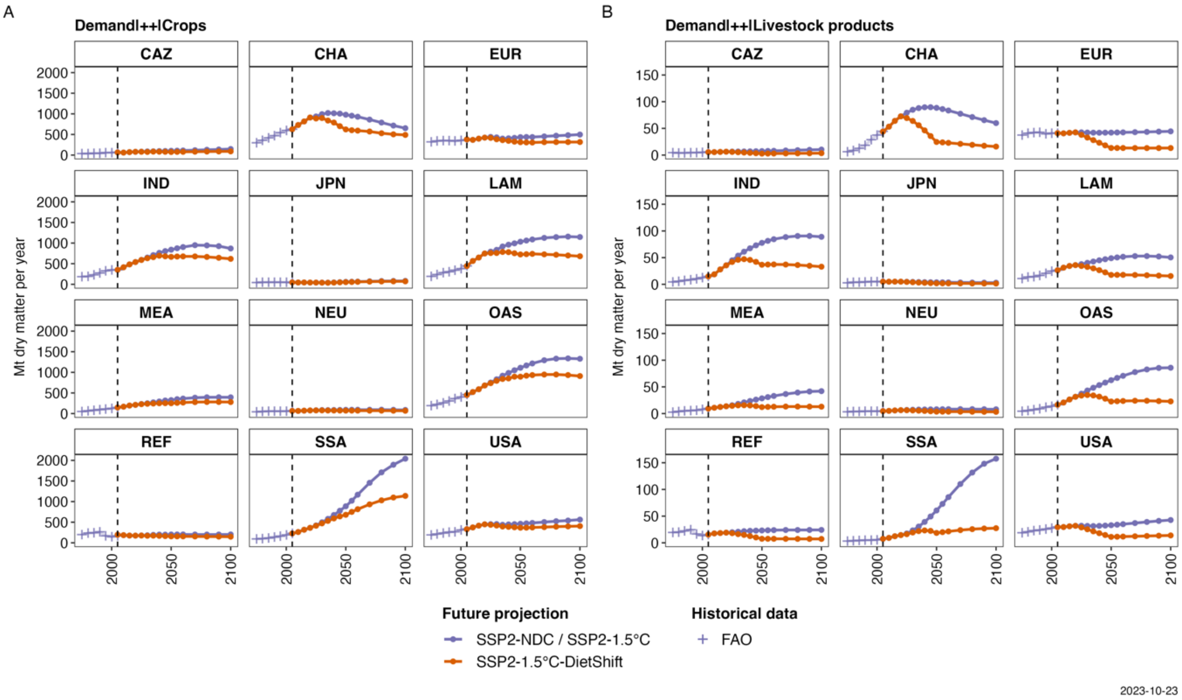

*Fig. S13: **Total agricultural demand for food, feed and other purposes.** Data is shown at regional level. A) shows crops. B) shows livestock products. Historical data for comparison from FAO (62). The historical data has been processed using the pik-piam/mrvalidation R package (59).*

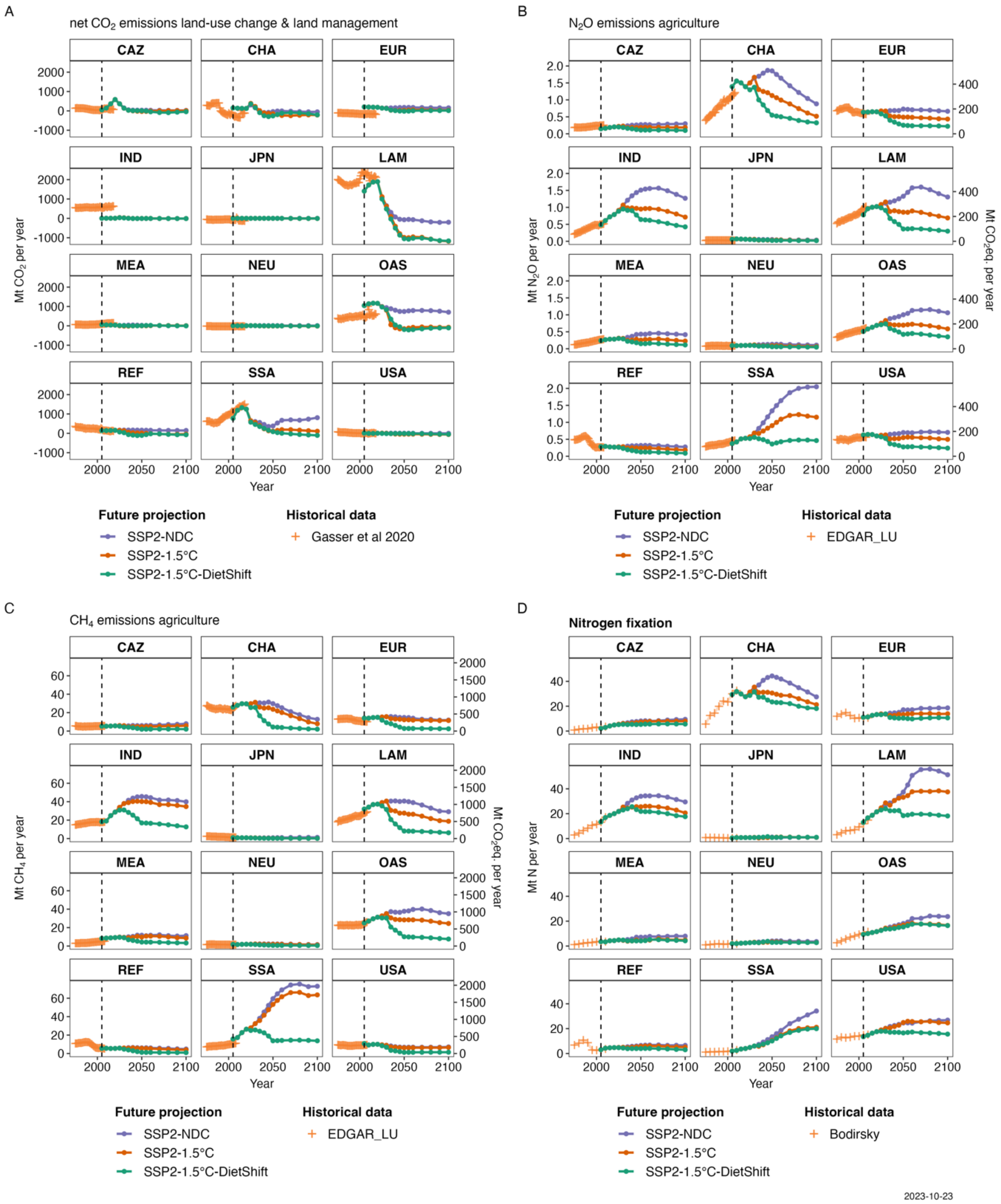

Fig. S14: ***Validation of environmental indicators: GHG emissions and nitrogen fixation.*** *Data is shown at regional level. A) shows net CO_2 emissions from land-use change and management. B) shows N_2O emissions from agriculture. C) shows CH_4 emissions from agriculture. D) shows nitrogen fixation. For the conversion of N_2O and CH_4 emissions into CO_2 equivalents (right axis) we used IPCC AR6 GWP100 factors of 273 and 27, respectively. Historical data for comparison from Gasser et al (63), the EDGAR emissions database version 4.2 (64) and Bodirsky et al (50). The historical data has been processed using the pik-piam/mrvalidation R package (59).*

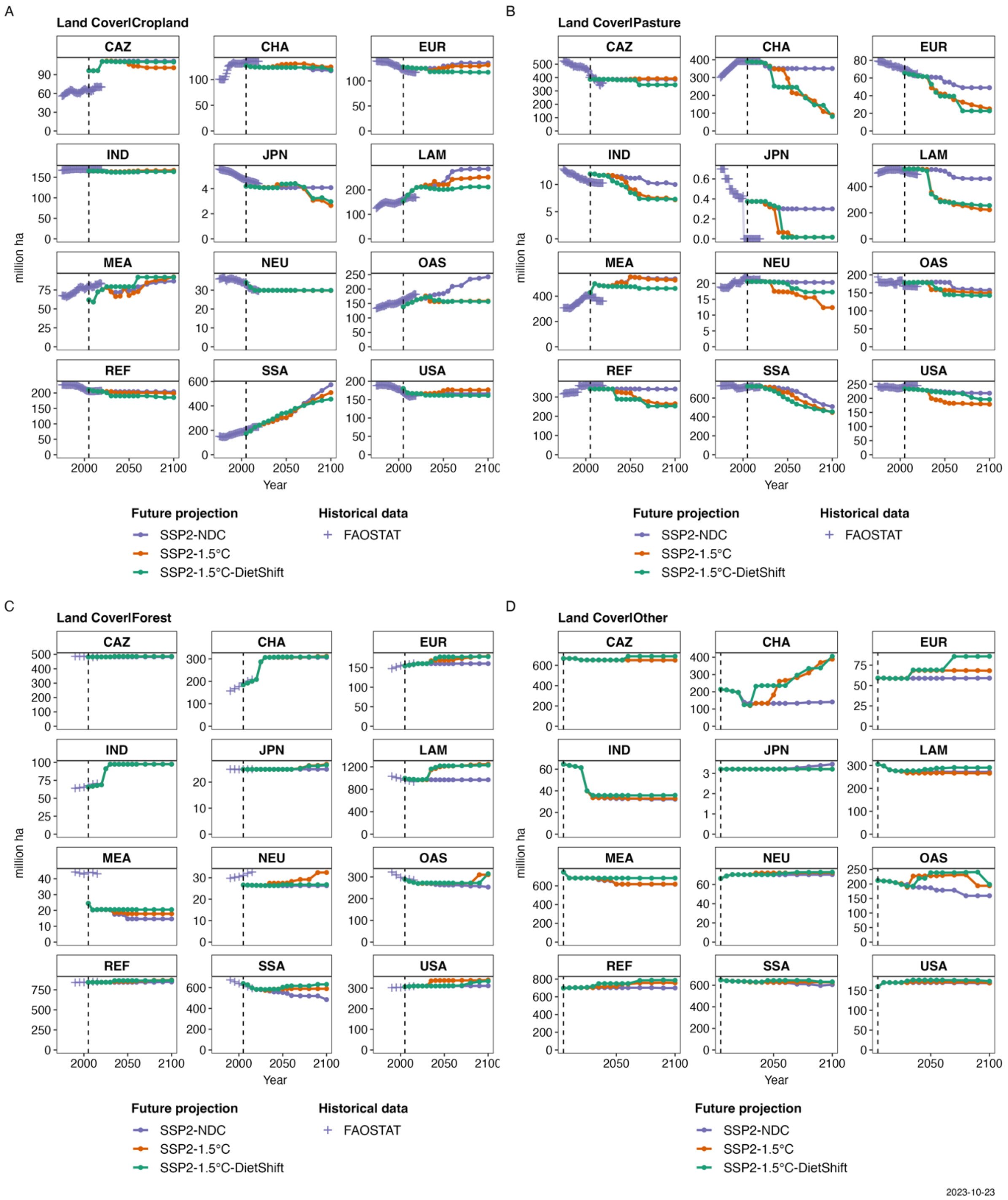

*Fig. S15: **Validation of main land classes.** Data is shown at regional level. A) shows cropland. B) shows pasture. C) shows forest. D) shows other natural land. Historical data for comparison from FAO (62).The historical data has been processed using the pik-piam/mrvalidation R package (59).*

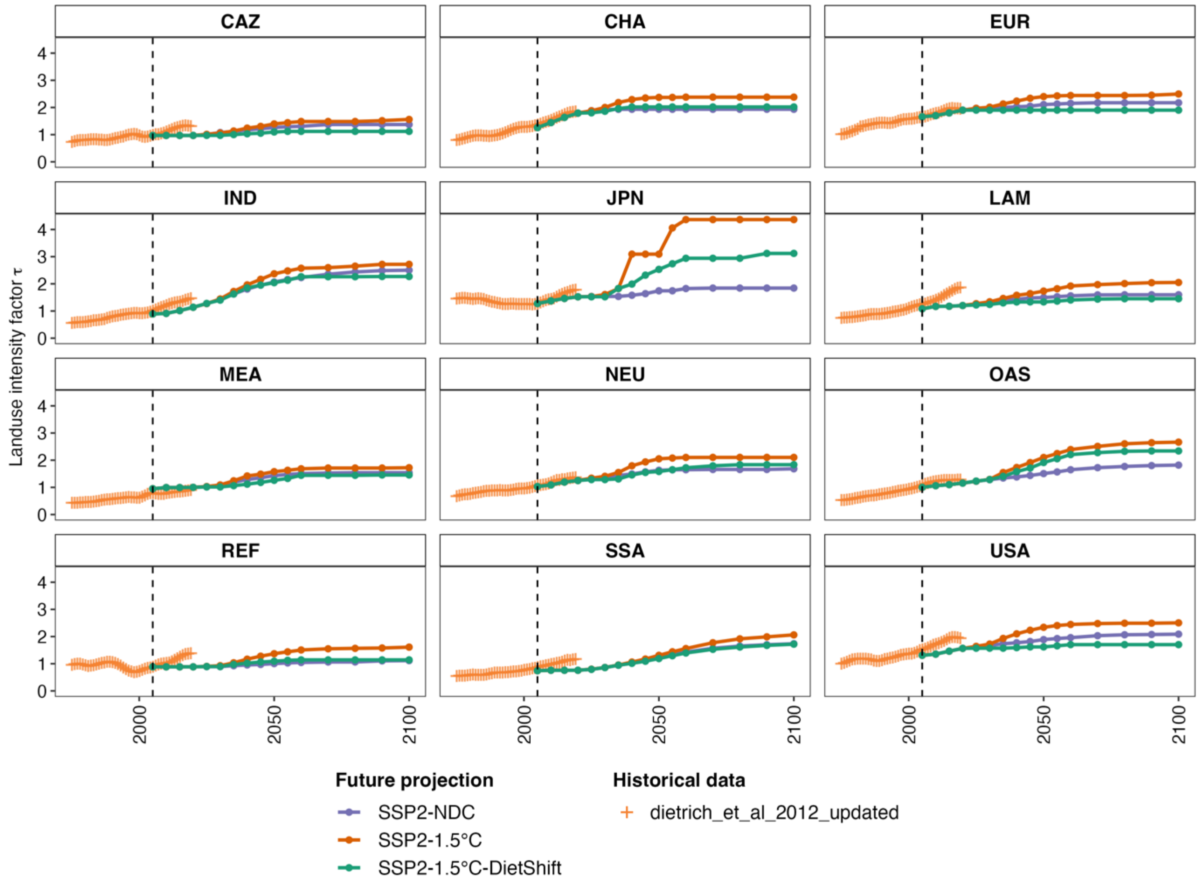

*Fig. S16: **Land-use intensity factor τ.** The τ factor reflects the degree of crop yield amplification caused by human activities. A duplication of τ implies a doubling of crop yields under fixed environmental conditions. Data is shown at regional level. Historical data for comparison from Dietrich et al (65). The historical data has been processed using the pik-piam/mrvalidation R package (59).*

REFERENCES AND NOTES

1. IPCC, “Summary for Policymakers” in Climate Change 2022: Mitigation of Climate Change. Contribution of Working Group III to the Sixth Assessment Report of the Intergovernmental Panel on Climate Change, P. R. Shukla, J. Skea, R. Slade, A. Al Khourdajie, R. van Diemen, D. McCollum, M. Pathak, S. Some, P. Vyas, R. Fradera, M. Belkacemi, A. Hasija, G. Lisboa, S. Luz, J. Malley, Eds. (Cambridge Univ. Press, USA, 2022).

2. W. Willett, J. Rockström, B. Loken, M. Springmann, T. Lang, S. Vermeulen, T. Garnett, D. Tilman, F. DeClerck, A. Wood, M. Jonell, M. Clark, L. J. Gordon, J. Fanzo, C. Hawkes, R. Zurayk, J. A. Rivera, W. De Vries, L. Majele Sibanda, A. Afshin, A. Chaudhary, M. Herrero, R. Agustina, F. Branca, A. Lartey, S. Fan, B. Crona, E. Fox, V. Bignet, M. Troell, T. Lindahl, S. Singh, S. E. Cornell, K. Srinath Reddy, S. Narain, S. Nishtar, C. J. L. Murray, Food in the anthropocene: The EAT–Lancet Commission on healthy diets from sustainable food systems. *Lancet* **393**, 447–492 (2019).

3. B. Soergel, E. Kriegler, I. Weindl, S. Rauner, A. Dirnaichner, C. Ruhe, M. Hofmann, N. Bauer, C. Bertram, B. L. Bodirsky, M. Leimbach, J. Leininger, A. Levesque, G. Luderer, M. Pehl, C. Wingens, L. Baumstark, F. Beier, J. P. Dietrich, F. Humpenöder, P. von Jeetze, D. Klein, J. Koch, R. Pietzcker, J. Strefler, H. Lotze-Campen, A. Popp, A sustainable development pathway for climate action within the UN 2030 Agenda. *Nat. Clim. Change* **11**, 656–664 (2021).

4. J. C. Doelman, F. D. Beier, E. Stehfest, B. L. Bodirsky, A. H. W. Beusen, F. Humpenöder, A. Mishra, A. Popp, D. P. van Vuuren, L. de Vos, I. Weindl, W.-J. van Zeist, T. Kram, Quantifying synergies and trade-offs in the global water-land-food-climate nexus using a multi-model scenario approach. *Environ. Res. Lett.* **17**, 045004 (2022).

5. Q. D. Read, K. L. Hondula, M. K. Muth, Biodiversity effects of food system sustainability actions from farm to fork. *Proc. Natl. Acad. Sci. U.S.A.* **119**, e2113884119 (2022).

6. M. Springmann, M. Clark, D. Mason-D’Croz, K. Wiebe, B. L. Bodirsky, L. Lassaletta, W. de Vries, S. J. Vermeulen, M. Herrero, K. M. Carlson, M. Jonell, M. Troell, F. DeClerck, L. J. Gordon, R. Zurayk, P. Scarborough, M. Rayner, B. Loken, J. Fanzo, H. C. J. Godfray, D. Tilman, J. Rockström, W. Willett, Options for keeping the food system within environmental limits. *Nature* **562**, 519–525 (2018).

7. E. Lucas, M. Guo, G. Guillén-Gosálbez, Low-carbon diets can reduce global ecological and health costs. *Nat. Food* **4**, 394–406 (2023).

8. S. Frank, P. Havlík, E. Stehfest, H. van Meijl, P. Witzke, I. Pérez-Domínguez, M. van Dijk, J. C. Doelman, T. Fellmann, J. F. L. Koopman, A. Tabeau, H. Valin, Agricultural non-CO2 emission reduction potential in the context of the 1.5 °C target. *Nat. Clim. Change* **9**, 66–72 (2019).

9. C. Costa Jr., E. Wollenberg, M. Benitez, R. Newman, N. Gardner, F. Bellone, Roadmap for achieving net-zero emissions in global food systems by 2050. *Sci. Rep.* **12**, 15064 (2022).

10. Z. Sun, L. Scherer, A. Tukker, S. A. Spawn-Lee, M. Bruckner, H. K. Gibbs, P. Behrens, Dietary change in high-income nations alone can lead to substantial double climate dividend. *Nat. Food* **3**, 29–37 (2022).

11. F. Humpenöder, A. Popp, C.-F. Schleussner, A. Orlov, M. G. Windisch, I. Menke, J. Pongratz, F. Havermann, W. Thiery, F. Luo, P. V. Jeetze, J. P. Dietrich, H. Lotze-Campen, I. Weindl, Q. Lejeune, Overcoming global inequality is critical for land-based mitigation in line with the Paris Agreement. *Nat. Commun.* **13**, 7453 (2022).

12. F. Humpenöder, B. L. Bodirsky, I. Weindl, H. Lotze-Campen, T. Linder, A. Popp, Projected environmental benefits of replacing beef with microbial protein. *Nature* **605**, 90–96 (2022).

13. B. L. Bodirsky, D. M.-C. Chen, I. Weindl, B. Soergel, F. Beier, E. J. Molina Bacca, F. Gaupp, A. Popp, H. Lotze-Campen, Integrating degrowth and efficiency perspectives enables an emission-neutral food system by 2100. *Nat. Food* **3**, 341–348 (2022).

14. A. Grubler, C. Wilson, N. Bento, B. Boza-Kiss, V. Krey, D. L. McCollum, N. D. Rao, K. Riahi, J. Rogelj, S. De Stercke, J. Cullen, S. Frank, O. Fricko, F. Guo, M. Gidden, P. Havlík, D. Huppmann, G. Kiesewetter, P. Rafaj, W. Schoepp, H. Valin, A low energy demand scenario for meeting the 1.5 °C target and sustainable development goals without negative emission technologies. *Nat. Energy* **3**, 515–527 (2018).

15. D. P. van Vuuren, E. Stehfest, D. E. H. J. Gernaat, M. van den Berg, D. L. Bijl, H. S. de Boer, V. Daioglou, J. C. Doelman, O. Y. Edelenbosch, M. Harmsen, A. F. Hof, M. A. E. van Sluisveld, Alternative pathways to the 1.5 °C target reduce the need for negative emission technologies. *Nat. Clim. Change* **8**, 391–397 (2018).

16. C. C. Ivanovich, T. Sun, D. R. Gordon, I. B. Ocko, Future warming from global food consumption. *Nat. Clim. Change* **13**, 297–302 (2023).

17. F. Gaupp, C. Ruggeri Laderchi, H. Lotze-Campen, F. DeClerck, B. L. Bodirsky, S. Lowder, A. Popp, R. Kanbur, O. Edenhofer, R. Nugent, J. Fanzo, S. Dietz, S. Nordhagen, S. Fan, Food system development pathways for healthy, nature-positive and inclusive food systems. *Nat. Food* **2**, 928–934 (2021).

18. T. Searchinger, R. Waite, C. Hanson, J. Ranganathan, E. Matthews, Creating a sustainable food future: A menu of solutions to feed nearly 10 billion people by 2050. World Resources Institute, Washington DC, USA (2019).

19. M. Meinshausen, J. Lewis, C. McGlade, J. Gütschow, Z. Nicholls, R. Burdon, L. Cozzi, B. Hackmann, Realization of Paris Agreement pledges may limit warming just below 2 °C. *Nature* **604**, 304–309 (2022).

20. C. Smith, Z. R. J. Nicholls, K. Armour, W. Collins, P. Forster, M. Meinshausen, M. D. Palmer, M. Watanabe, The Earth's Energy Budget, Climate Feedbacks, Climate Sensitivity Supplementary Material, V. Masson-Delmotte, P. Zhai, A. Pirani, S. L. Connors, C. Péan, S. Berger, N. Caud, Y. Chen, L. Goldfarb, M. I. Gomis, M. Huang, K. Leitzell, E. Lonnoy, J. B. R. Matthews, T. K. Maycock, T. Waterfield, O. Yelekçi, R. Yu, B. Zhou, Eds., Climate Change 2021: The Physical Science Basis. Contribution of Working Group I to the Sixth Assessment Report of the Intergovernmental Panel on Climate Change (IPCC, 2021); www.ipcc.ch/.

21. C. Béné, J. Fanzo, L. Haddad, C. Hawkes, P. Caron, S. Vermeulen, M. Herrero, P. Oosterveer, Five priorities to operationalize the EAT–Lancet Commission report. *Nat. Food* **1**, 457–459 (2020).

22. F. Funke, L. Mattauch, I. van den Bijgaart, C. Godfray, C. J. Hepburn, D. Klenert, M. Springmann, N. Treich, Is meat too cheap? Towards optimal meat taxation. *SSRN Electron. J.* 10.2139/ssrn.3801702 (2021).

23. C. Latka, M. Kuiper, S. Frank, T. Heckelei, P. Havlík, H.-P. Witzke, A. Leip, H. D. Cui, A. Kuijsten, J. M. Geleijnse, M. Van Dijk, Paying the price for environmentally sustainable and healthy EU diets. *Glob. Food Secur.* **28**, 100437 (2021).

24. A. Muhammad, J. L. Seale Jr., B. Meade, A. Regmi, International Evidence on Food Consumption Patterns: An Update Using 2005 Internation Comparison Program Data. *SSRN Electron. J.* 10.2139/ssrn.2114337 (2011).

25. D. Klenert, F. Funke, M. Cai, Meat taxes in Europe can be designed to avoid overburdening low-income consumers. *Nat. Food* **4**, 894–901 (2023).

26. F. Femenia, A meta-analysis of the price and income elasticities of food demand. *Ger. J. Agric. Econ.* **68**, 10.22004/ag.econ.319809 (2019).

27. C. A. Gallet, Meat meets meta: A quantitative review of the price elasticity of meat. *Am. J. Agric. Econ.* **92**, 258–272 (2010).

28. T. Andreyeva, M. W. Long, K. D. Brownell, The impact of food prices on consumption: A systematic review of research on the price elasticity of demand for food. *Am. J. Public Health* **100**, 216–222 (2010).

29. J. Poore, T. Nemecek, Reducing food's environmental impacts through producers and consumers. *Science* **360**, 987–992 (2018).

30. T. Kwasny, K. Dobernig, P. Riefler, Towards reduced meat consumption: A systematic literature review of intervention effectiveness, 2001–2019. *Appetite* **168**, 105739 (2022).

31. K. Parsons, "Who makes food policy in England? A Map of Government Actors and Activities" (Food Research Collaboration, 2020).

32. L. P. Fesenfeld, M. Wicki, Y. Sun, T. Bernauer, Policy packaging can make food system transformation feasible. *Nat. Food* **1**, 173–182 (2020).

33. M. Springmann, M. A. Clark, M. Rayner, P. Scarborough, P. Webb, The global and regional costs of healthy and sustainable dietary patterns: A modelling study. *Lancet Planet. Health* **5**, e797–e807 (2021).

34. M. Springmann, D. Mason-D'Croz, S. Robinson, T. Garnett, H. C. J. Godfray, D. Gollin, M. Rayner, P. Ballon, P. Scarborough, Global and regional health effects of future food production under climate change: A modelling study. *Lancet* **387**, 1937–1946 (2016).

35. D. I. Armstrong McKay, A. Staal, J. F. Abrams, R. Winkelmann, B. Sakschewski, S. Loriani, I. Fetzer, S. E. Cornell, J. Rockström, T. M. Lenton, Exceeding 1.5°C global warming could trigger multiple climate tipping points. *Science* **377**, eabn7950 (2022).

36. J. G. Canadell, P. M. S. Monteiro, M. H. Costa, L. Cotrim da Cunha, P. M. Cox, A. V. Eliseev, S. Henson, M. Ishii, S. Jaccard, C. Koven, A. Lohila, P. K. Patra, S. Piao, J. Rogelj, S. Syampungani, S. Zaehle, K. Zickfeld, Global Carbon and other Biogeochemical Cycles and Feedbacks, V. Masson-Delmotte, P. Zhai, A. Pirani, S. L. Connors, C. Péan, S. Berger, N. Caud, Y. Chen, L. Goldfarb, M. I. Gomis, M. Huang, K. Leitzell, E. Lonnoy, J. B. R. Matthews, T. K. Maycock, T. Waterfield, O. Yelekçi, R. Yu, B. Zhou, Eds., Climate Change 2021: The Physical Science Basis. Contribution of Working Group I to the Sixth Assessment Report of the Intergovernmental Panel on Climate Change (IPCC, 2021), pp. 673–816.

37. N. Bauer, D. Klein, F. Humpenöder, E. Kriegler, G. Luderer, A. Popp, J. Strefler, Bio-energy and CO2 emission reductions: An integrated land-use and energy sector perspective. *Clim. Change* **163**, 1675–1693 (2020).

38. M. Meinshausen, T. M. L. Wigley, S. C. B. Raper, Emulating atmosphere-ocean and carbon cycle models with a simpler model, MAGICC6 – Part 2: Applications. *Atmos. Chem. Phys.* **11**, 1457–1471 (2011).

39. E. Byers, V. Krey, E. Kriegler, K. Riahi, R. Schaeffer, J. Kikstra, R. Lamboll, Z. Nicholls, M. Sandstad, C. Smith, K. van der Wijst, F. Lecocq, J. Portugal-Pereira, Y. Saheb, A. Stromann, H. Winkler, C. Auer, E. Brutschin, C. Lepault, E. Müller-Casseres, M. Gidden, D. Huppmann, P. Kolp, G. Marangoni, M. Werning, K. Calvin, C. Guivarch, T. Hasegawa, G. Peters, J. Steinberger, M. Tavoni, D. van Vuuren, A. Al -Khourdajie, P. Forster, J. Lewis, M.

Meinshausen, J. Rogelj, B. Samset, R. Skeie, IPCC AR6 Scenarios Database, version 1.0 (Zenodo, 2022); https://doi.org/10.5281/zenodo.5886912.

40. G. Luderer, S. Madeddu, L. Merfort, F. Ueckerdt, M. Pehl, R. Pietzcker, M. Rottoli, F. Schreyer, N. Bauer, L. Baumstark, C. Bertram, A. Dirnaichner, F. Humpenöder, A. Levesque, A. Popp, R. Rodrigues, J. Strefler, E. Kriegler, Impact of declining renewable energy costs on electrification in low-emission scenarios. *Nat. Energy* **7**, 32–42 (2022).

41. G. Luderer, N. Bauer, L. Baumstark, C. Bertram, M. Leimbach, R. Pietzcker, J. Strefler, T. Aboumahboub, G. Abrahão, C. Auer, F. Benke, S. Bi, J. Dietrich, A. Dirnaichner, S. Fuchs, P. Führlich, A. Giannousakis, C. C. Gong, M. Haller, R. Hasse, J. Hilaire, J. Hoppe, D. Klein, J. Koch, A. Körner, K. Kowalczyk, E. Kriegler, A. Levesque, A. Lorenz, S. Ludig, M. Lüken, A. Malik, R. Mandaroux, S. Manger, A. Merfort, L. Merfort, S. Moreno-Leiva, I. Mouratiadou, A. Odenweller, M. Pehl, M. Pflüger, F. Piontek, L. Popin, S. Rauner, O. Richters, R. Rodrigues, N. Roming, M. Rottoli, E. Schmidt, C. Schötz, F. Schreyer, A. Schultes, B. Sörgel, F. Ueckerdt, P. Verpoort, P. Weigmann, REMIND - REgional Model of INvestments and Development - version 3.2.0 (Zenodo, 2023); https://github.com/remindmodel, https://doi.org/10.5281/zenodo.7852740.

42. J. P. Dietrich, B. L. Bodirsky, F. Humpenöder, I. Weindl, M. Stevanović, K. Karstens, U. Kreidenweis, X. Wang, A. Mishra, D. Klein, G. Ambrósio, E. Araujo, A. W. Yalew, L. Baumstark, S. Wirth, A. Giannousakis, F. Beier, D. M.-C. Chen, H. Lotze-Campen, A. Popp, MAgPIE 4—A modular open-source framework for modeling global land systems. *Geosci. Model Dev.* **12**, 1299–1317 (2019).

43. J. P. Dietrich, B. L. Bodirsky, I. Weindl, F. Humpenöder, M. Stevanovic, U. Kreidenweis, X. Wang, K. Karstens, A. Mishra, F. D. Beier, E. J. M. Bacca, P. von Jeetze, M. Windisch, M. S. Crawford, D. Leip, D. Klein, V. Singh, G. Ambrósio, E. Araujo, A. Biewald, P. Führlich, H.

Lotze-Campen, A. Popp, MAgPIE - An Open Source land-use modeling framework version 4.6.7 (Zenodo, 2023); https://github.com/magpiemodel, https://doi.org/10.5281/zenodo.7923602.

44. S. Schaphoff, W. von Bloh, A. Rammig, K. Thonicke, H. Biemans, M. Forkel, D. Gerten, J. Heinke, J. Jägermeyr, J. Knauer, F. Langerwisch, W. Lucht, C. Müller, S. Rolinski, K. Waha, LPJmL4—A dynamic global vegetation model with managed land—Part 1: Model description. *Geosci. Model Dev.* **11**, 1343–1375 (2018).

45. W. von Bloh, S. Schaphoff, C. Müller, S. Rolinski, K. Waha, S. Zaehle, Implementing the nitrogen cycle into the dynamic global vegetation, hydrology, and crop growth model LPJmL (version 5.0). *Geosci. Model Dev.* **11**, 2789–2812 (2018).

46. T. Herzfeld, J. Heinke, S. Rolinski, C. Müller, Soil organic carbon dynamics from agricultural management practices under climate change. *Earth Syst. Dynam.* **12**, 1037–1055 (2021).

47. F. Lutz, S. Del Grosso, S. Ogle, S. Williams, S. Minoli, S. Rolinski, J. Heinke, J. J. Stoorvogel, C. Müller, The importance of management information and soil moisture representation for simulating tillage effects on N2O emissions in LPJmL5.0-tillage. *Geosci. Model Dev.* **13**, 3905–3923 (2020).

48. J. P. Dietrich, C. Schmitz, H. Lotze-Campen, A. Popp, C. Müller, Forecasting technological change in agriculture—An endogenous implementation in a global land use model. *Technol. Forecast. Soc. Change* **81**, 236–249 (2014).

49. M. Stevanović, A. Popp, B. L. Bodirsky, F. Humpenöder, C. Müller, I. Weindl, J. P. Dietrich, H. Lotze-Campen, U. Kreidenweis, S. Rolinski, A. Biewald, X. Wang, Mitigation strategies for greenhouse gas emissions from agriculture and land-use change: Consequences for food prices. *Environ. Sci. Technol.* **51**, 365–374 (2017).

50. B. L. Bodirsky, A. Popp, H. Lotze-Campen, J. P. Dietrich, S. Rolinski, I. Weindl, C. Schmitz, C. Müller, M. Bonsch, F. Humpenöder, A. Biewald, M. Stevanovic, Reactive nitrogen requirements to feed the world in 2050 and potential to mitigate nitrogen pollution. *Nat. Commun.* **5**, 3858 (2014).

51. A. Popp, H. Lotze-Campen, B. Bodirsky, Food consumption, diet shifts and associated non-CO2 greenhouse gases from agricultural production. *Glob. Environ. Change* **20**, 451–462 (2010).

52. F. Humpenöder, K. Karstens, H. Lotze-Campen, J. Leifeld, L. Menichetti, A. Barthelmes, A. Popp, Peatland protection and restoration are key for climate change mitigation. *Environ. Res. Lett.* **15**, 104093 (2020).

53. K. Riahi, D. P. van Vuuren, E. Kriegler, J. Edmonds, B. C. O'Neill, S. Fujimori, N. Bauer, K. Calvin, R. Dellink, O. Fricko, W. Lutz, A. Popp, J. C. Cuaresma, S. Kc, M. Leimbach, L. Jiang, T. Kram, S. Rao, J. Emmerling, K. Ebi, T. Hasegawa, P. Havlik, F. Humpenöder, L. A. Da Silva, S. Smith, E. Stehfest, V. Bosetti, J. Eom, D. Gernaat, T. Masui, J. Rogelj, J. Strefler, L. Drouet, V. Krey, G. Luderer, M. Harmsen, K. Takahashi, L. Baumstark, J. C. Doelman, M. Kainuma, Z. Klimont, G. Marangoni, H. Lotze-Campen, M. Obersteiner, A. Tabeau, M. Tavoni, The Shared Socioeconomic Pathways and their energy, land use, and greenhouse gas emissions implications: An overview. *Glob. Environ. Change* **42**, 153–168 (2017).

54. A. Popp, K. Calvin, S. Fujimori, P. Havlik, F. Humpenöder, E. Stehfest, B. L. Bodirsky, J. P. Dietrich, J. C. Doelmann, M. Gusti, T. Hasegawa, P. Kyle, M. Obersteiner, A. Tabeau, K. Takahashi, H. Valin, S. Waldhoff, I. Weindl, M. Wise, E. Kriegler, H. Lotze-Campen, O. Fricko, K. Riahi, D. P. van Vuuren, Land-use futures in the shared socio-economic pathways. *Glob. Environ. Change* **42**, 331–345 (2017).

55. L. Baumstark, N. Bauer, F. Benke, C. Bertram, S. Bi, C. C. Gong, J. P. Dietrich, A. Dirnaichner, A. Giannousakis, J. Hilaire, D. Klein, J. Koch, M. Leimbach, A. Levesque, S. Madeddu, A. Malik, A. Merfort, L. Merfort, A. Odenweller, M. Pehl, R. C. Pietzcker, F. Piontek, S. Rauner, R. Rodrigues, M. Rottoli, F. Schreyer, A. Schultes, B. Soergel, D. Soergel, J. Strefler, F. Ueckerdt, E. Kriegler, G. Luderer, REMIND2.1: Transformation and innovation dynamics of the energy-economic system within climate and sustainability limits. *Geosci. Model Dev.* **14**, 6571–6603 (2021).

56. M. Harmsen, C. Tabak, L. Höglund-Isaksson, F. Humpenöder, P. Purohit, D. van Vuuren, Uncertainty in non-CO2 greenhouse gas mitigation contributes to ambiguity in global climate policy feasibility. *Nat. Commun.* **14**, 2949 (2023).

57. S. Kc, W. Lutz, The human core of the shared socioeconomic pathways: Population scenarios by age, sex and level of education for all countries to 2100. *Glob. Environ. Change* **42**, 181–192 (2017).

58. World Bank, "World Development Indicators" (2019); https://databank.worldbank.org.

59. B. L. Bodirsky, S. Wirth, K. Karstens, F. Humpenoeder, M. Stevanovic, A. Mishra, A. Biewald, I. Weindl, F. Beier, D. Chen, M. Crawford, D. Leip, E. M. Bacca, U. Kreidenweis, A. W. Yalew, F. Humpenoeder, P. von Jeetze, X. Wang, J. P. Dietrich, M. Alves, Mrvalidation: Madrat data preparation for validation purposes (Zenodo, 2022); https://doi.org/10.5281/zenodo.4317826.

60. R. Dellink, J. Chateau, E. Lanzi, B. Magné, Long-term economic growth projections in the Shared Socioeconomic Pathways. *Glob. Environ. Change* **42**, 200–214 (2017).

61. S. L. James, P. Gubbins, C. J. Murray, E. Gakidou, Developing a comprehensive time series of GDP per capita for 210 countries from 1950 to 2015. *Popul. Health Metr.* **10**, 12 (2012).

62. FAO, “FAOSTAT” (Food and Agriculture Organization of the United Nations, 2021).

63. T. Gasser, L. Crepin, Y. Quilcaille, R. A. Houghton, P. Ciais, M. Obersteiner, Historical CO2 emissions from land use and land cover change and their uncertainty. *Biogeosciences* **17**, 4075–4101 (2020).

64. European Commission, Joint Research Centre (JRC)/Netherlands Environmental Assessment Agency (PBL), “Emission Database for Global Atmospheric Research (EDGAR), release version 4.2” (2011); http://edgar.jrc.ec.europa.eu.

65. J. P. Dietrich, C. Schmitz, C. Müller, M. Fader, H. Lotze-Campen, A. Popp, Measuring agricultural land-use intensity – A global analysis using a model-assisted approach. *Ecol. Model.* **232**, 109–118 (2012).
